# Longitudinal Awake Mouse Brain Imaging Using Functional Ultrasound and Functional Ultrasound Localization Microscopy

**DOI:** 10.1002/advs.77931

**Published:** 2026-09-27

**Authors:** Zhe Huang, Yike Wang, Matthew R. Lowerison, Yue Xu, Murong Yi, Bing‐Ze Lin, YiRang Shin, Nathiya Vaithiyalingam Chandra Sekaran, Daniel A. Llano, Pengfei Song

**Affiliations:** ^1^ Department of Biomedical Engineering Duke University Durham North Carolina USA; ^2^ Department of Electrical and Computer Engineering University of Illinois Urbana‐Champaign Urbana Illinois USA; ^3^ Beckman Institute for Advanced Science and Technology University of Illinois at Urbana‐Champaign Urbana Illinois USA; ^4^ Neuroscience Program University of Illinois at Urbana‐Champaign Urbana Illinois USA

**Keywords:** Awake imaging, cerebrovascular imaging, functional ultrasound, functional ultrasound localization microscopy, longitudinal imaging, ultrasound localization microscopy

## Abstract

Functional ultrasound (fUS), ultrasound localization microscopy (ULM), and functional ULM (fULM) are three emerging brain ultrasound imaging techniques that measure the cerebrovascular structure and function at different spatial scales. Here we establish an awake, longitudinal fUS‐ULM‐fULM imaging framework to study structural vascularity, microvascular flow velocity, mesoscale hemodynamic responses, and microvascular functional responses in the same animals across 5 months. No significant linear longitudinal trend was detected for any of the ultrasound measurements in control animals, with structural measures showing lower longitudinal variability than functional readouts. Mean flow velocity showed the strongest longitudinal consistency and the lowest pooled within‐subject variability. Behavioral testing and GFAP/Iba1 staining further revealed no detectable memory impairment or chronic neuroinflammatory response during the 5‐month serial imaging setup. Application of fUS‐ULM‐fULM in 5xFAD‐positive mice revealed a temporally ordered, cross‐scale pattern of cerebrovascular dysfunction, with cerebral blood flow velocity declining first followed by impaired stimulus‐evoked responses at the microvascular and then mesoscale levels. No vascular structural changes were detected across 5 months. Overall, this study established a stable longitudinal multimodal ultrasound imaging framework for tracking the awake mouse brain and demonstrated the utility for detecting cerebrovascular structural and functional variations in a preclinical Alzheimer's disease mouse model.

## Graphical abstract

Awake longitudinal multimodal ultrasound imaging integrates functional ultrasound (fUS), ultrasound localization microscopy (ULM), and functional ULM (fULM) to characterize cerebrovascular function, microvascular architecture, and flow dynamics within the same animals over time.

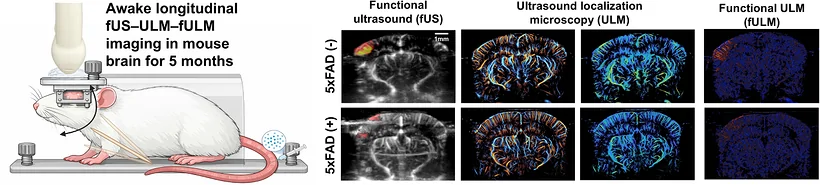

**Abbreviations**
CBF: cerebral blood flow
fUS: functional ultrasound
ULM: ultrasound localization microscopy
fULM: functional ultrasound localization microscopy
CV: coefficient of variation
ICC: intraclass correlation coefficient
RC: repeatability coefficient;
MDC_95_
: minimal detectable change at the 95% confidence level
SVD: singular value decomposition
GLM: general linear model
ROI: region of interest
PSF: point spread function
IQ: in‐phase quadrature

## 1 Introduction

Many neurological and neurodegenerative disorders, most notably Alzheimer's Disease and Vascular Cognitive Impairment, are closely associated with abnormalities of the cerebral vasculature, including dysregulated blood flow, vascular remodeling, and impaired neurovascular coupling [1, 2]. Gaining mechanistic insight into these diseases and developing effective therapeutic strategies rely heavily on mouse models, which enable causal and longitudinal investigation of disease processes under well‐defined experimental conditions. In this context, advanced neuroimaging is an essential tool for interrogating such abnormalities in the in vivo mouse brain. By monitoring the long‐term evolution of the cerebral vasculature and characterizing neurovascular homeostasis across physiological and pathological states, such imaging technologies provide a critical foundation for understanding the onset and progression of neurological and neurodegenerative disorders as well as the effectiveness of therapies and interventions.

To meet this requirement, imaging approaches capable of simultaneously capturing large‐scale brain territories and fine‐scale local regions are essential [3]. Although existing neuroimaging techniques such as magnetic resonance imaging (MRI) [4, 5] and optical imaging [6, 7] have substantially advanced our understanding of the brain, they face inherent limitations in the context of imaging territory and spatial resolution. MRI provides whole‐brain coverage but lacks the spatial resolution and specificity to resolve microscopic vasculature and micro‐circuitry‐level brain activity [4, 5]. In contrast, optical imaging provides exquisite microscopic resolution but suffers from limited imaging depth [6, 7], which confines its utility to superficial brain regions. In recent years, ultrasound has rapidly emerged as a promising brain imaging modality [8] that bridges the imaging resolution and coverage gap between MRI and optics by using high frequency ultrasound waves that provide both robust imaging depth of penetration and spatial resolution. At present, three major ultrasound brain imaging techniques are used for studying the rodent brain: Functional ultrasound (fUS) is a contrast‐free brain blood flow imaging technique that is sensitive to functional hyperemia and provides maps of cerebral blood volume changes (ΔCBV) across brain‐wide territories at a mesoscopic spatial resolution [9]. Ultrasound localization microscopy (ULM) is a contrast‐enhanced super‐resolution imaging approach that localizes and tracks circulating microbubbles (MBs) in the blood stream to visualize the microvascular architecture and measure their flow [10, 11]. ULM also allows detailed assessments of local and global vessel morphology (e.g., density, tortuosity, intervessel distance) and flow distributions [12, 13, 14]; functional ULM (fULM) is also a contrast‐enhanced imaging technique that essentially combines ULM and fUS, which enables microvessel‐scale mapping of cerebral blood flow (CBF) dynamics to detect stimulus‐locked hemodynamic responses manifested as MB count fluctuations modulated by the underlying neural activity [15]. Although all three ultrasound imaging modalities rely on blood flow measurements, they differ in functionality and provide complementary information about the brain. For example, both fUS and fULM provide functional mapping of the brain but they differ greatly in spatial (hundreds of microns for fUS, several microns for fULM) and temporal resolution (<1s for fUS and sub‐minute timescales for fULM). ULM complements fUS and fULM by serving as a primary structural imaging modality that reveals vascular anatomy and flow distributions. Therefore, integrating fUS, ULM, and fULM into a unified imaging platform will enable comprehensive structural and functional evaluations of the brain across different scales from the whole brain to individual microvessels, providing a powerful tool for studying neurological diseases in rodent models.

A major advantage of ultrasound‐based brain imaging is its compatibility with awake preparations, which is essential for studying brain function under naturalistic conditions without the confounding effects of anesthesia (e.g., isoflurane, which markedly alters CBF [16]). fUS has been implemented in both head‐fixed awake and freely moving animals, whereas ULM and fULM are currently more commonly performed in awake, head‐fixed preparations because they require stable probe positioning, prolonged acquisition, motion control, and consistent microbubble administration [17]. In addition, ultrasound is inherently well‐suited for longitudinal and serial imaging studies because of the lack of ionizing radiation and low cost [17]. Despite these advantages, however, fUS, ULM, and fULM have primarily been implemented independently in awake or longitudinal imaging paradigms [14] and they have not been combined within the same animals in a longitudinal setting to provide an integrated assessment of brain structure and function. Achieving such integration is technically nontrivial as it requires carefully coordinated acquisition protocols, imaging sequences, as well as stable and consistent microbubble administration in awake animals.

To this end, in this study we directly address these challenges by establishing an integrated fUS‐ULM‐fULM imaging platform for awake mouse brain imaging over time. We conducted a 5‐month longitudinal brain imaging study in awake mice, incorporating whisker stimulation during fUS and fULM to probe evoked functional vascular responses, while ULM was used to quantify microvascular structure and flow‐related metrics. We also evaluated chronic‐window safety and, to confirm that memory function remained intact after repeated imaging, assessed behavior using a passive avoidance task. In control mice, we first characterized the baseline variability, longitudinal repeatability, and modality‐specific detection thresholds of structural, flow‐related, and functional vascular measurements. We then applied the same imaging framework and baseline‐derived reference thresholds to a 5xFAD‐positive cohort to determine whether multimodal integration could detect disease‐related neurovascular alterations beyond normal longitudinal variability. By combining longitudinal stability assessment, pathological validation, and cross‐modal analyses, this study establishes a quantitative framework for distinguishing subtle disease‐associated neurovascular dysfunction from baseline fluctuations, and reveals how microvascular flow impairment, microscale functional deficits, and mesoscale hemodynamic responses are temporally and mechanistically related during early disease progression.

## 2 Results

**FIGURE 1:**
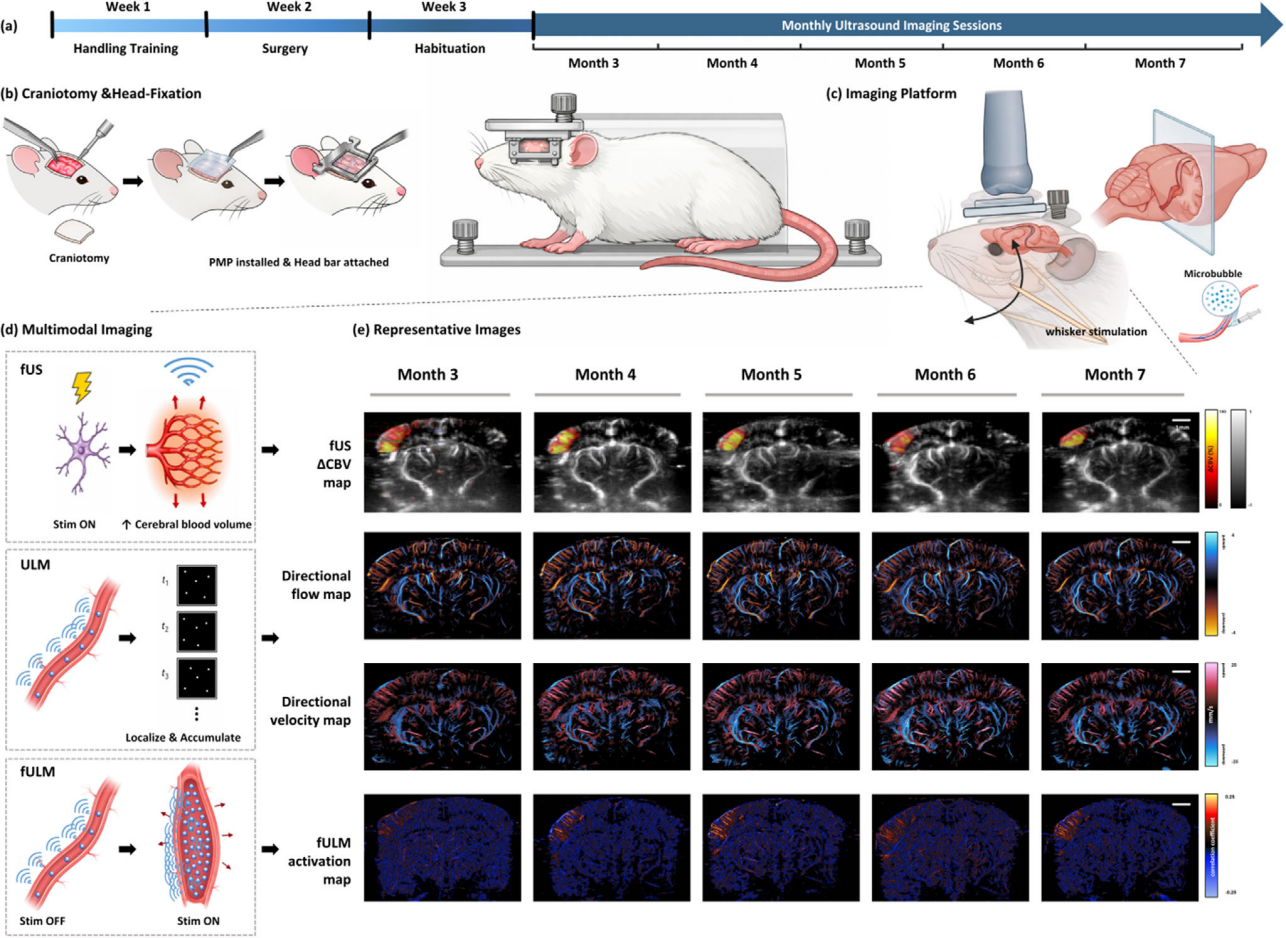
Schematic overview of the longitudinal awake ultrasound imaging workflow. (a) Experimental timeline showing handling training in Week 1, cranial window/head‐bar surgery in Week 2, habituation in Week 3, and monthly ultrasound imaging from Month 3 to Month 7. (b–d) Schematic illustrations of the craniotomy and head‐fixation preparation, awake imaging platform with whisker stimulation and microbubble injection, and the multimodal imaging strategy integrating fUS, ULM, and fULM. (e) Representative longitudinal images acquired from the same awake mice across five monthly sessions. Whisker‐evoked fUS ΔCBV maps show mesoscale hemodynamic responses in the S1 barrel cortex; ULM directional flow and directional velocity maps reveal microvascular architecture and flow dynamics; and fULM activation maps capture stimulus‐evoked microvascular functional responses. Created in BioRender. Lab, S. (2026) https://BioRender.com/w1cs5bc.

14 male mice were included in this study. Only male mice were used to reduce potential biological variability associated with sex‐dependent vascular and physiological fluctuations, including those related to the estrus cycle [18]. Each mouse was implanted with a transparent polymethylpentene (PMP) cranial window and allowed to recover for 14 days before imaging. Before cranial‐window and headpost implantation, the mean body weight of the 5xFAD‐negative mice was 23.8  ±  1.5 g. After recovery, the animal was placed in a custom‐designed body tube that was securely fixed to the recording table [19] (Figure 1a,b). The ultrasound probe was positioned above the brain and coupled with ultrasound gel to ensure optimal acoustic transmission (Figure 1c). Each mouse underwent longitudinal imaging (Figure 1d) that included (in the following order) whisker‐stimulation‐based fUS (Figure 1e, top row), ULM (Figure 1e, second and third rows), and whisker‐stimulation‐based fULM (Figure 1e, bottom row). Each animal was imaged over a five‐month period, with one imaging session conducted per month. Representative ultrasound images from one animal are presented in Figure 1e. A paired anesthetized‐versus‐awake comparison further supported the use of awake imaging for preserving functional hemodynamic responses (Figure S1a,b). After completion of the longitudinal imaging, the mice underwent behavioral testing and histological staining.

### 2.1 fUS Consistently Measures Mesoscale Brain Functional Responses to Whisker Stimulation

**FIGURE 2:**
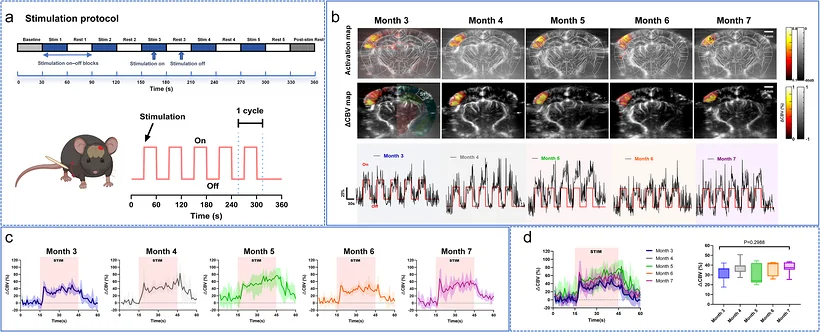
Longitudinal whisker‐stimulation‐based fUS imaging in awake mice. (a) The fUS imaging session lasted a total of 360 s, consisting of 30 s of baseline recording, five stimulation on‐off blocks (30 s of stimulation on followed by 30 s of stimulation off per block), and 30 s of post‐stimulation recovery in the end. (b) Representative fUS activation maps (top row, obtained from correlation between power Doppler signal and stimulus pattern), ΔCBV maps (middle row, obtained from calculating power Doppler signal difference between stimulation on and off), and ΔCBV time series (bottom row) from the same animal imaged longitudinally from 3 to 7 months of age. (c) Averaged ΔCBV time courses within the S1 region, each curve representing one monthly imaging session. For each month, the ΔCBV response was averaged across five stimulation cycles (one cycle defined as the stimulation on/off block shown in panel (b), lower‐left). Data are presented as mean (solid lines) ± SEM (shaded area). For this representative mouse, the mean ΔCBV across the five sessions was 42.28%, with a within‐mouse CV of 20.5%. (d) Group‐level comparison of mean ΔCBV across five monthly sessions (*n* = 7); one‐way ANOVA, *p* = 0.2988. Data are shown as box‐and‐whisker plots (median and IQR). fUS, functional ultrasound; ΔCBV, change in cerebral blood volume; SEM, standard error of the mean; IQR, interquartile range.

We characterized whisker stimulation‐evoked functional hyperemia in awake mice from 3 to 7 months of age using fUS. Across all longitudinal imaging sessions, whisker stimulation consistently elicited robust hemodynamic activation in the contralateral primary somatosensory cortex (S1), with significant ΔCBV response consistently localized to the barrel field (Figure 2a–c). No stimulus‐locked ΔCBV changes were observed in ipsilateral S1 or non‐sensory cortical regions, which confirms the spatial specificity of the response. Using mouse brain atlas [20], stimulus‐evoked functional activation remained localized to a similar cortical region over the five‐month imaging period. To quantify within‐mouse longitudinal spatial longitudinal repeatability, we used month 3 as the reference session and calculated the Jaccard index between the binarized activation map at month 3 and those at months 4, 5, 6, and 7. Across the seven mice, the mean Jaccard indices were 0.78 ± 0.11, 0.82 ± 0.09, 0.85 ± 0.13, and 0.81 ± 0.15, respectively, supporting stable spatial overlap of the evoked response across sessions. Quantitative analysis of ΔCBV time courses showed that the magnitude of the whisker stimulation responses exhibited no significant month‐to‐month variations (one‐way ANOVA, *p* = 0.2988; Figure 2d), with response amplitudes maintained within a range of 30%–38%. fUS ΔCBV had a pooled within‐subject CV of 15.4%, an intraclass correlation coefficient (ICC) of 0.47, a repeatability coefficient (RC) of 15.1%, and no significant longitudinal trend (linear mixed‐effects models, trend *p* = 0.306; Table 1), indicating moderate session‐to‐session variability without evidence of systematic drift over time. Paired TOST analysis supported equivalence between Month 3 and Month 7, with a mean difference of 4.27% and a 90% confidence interval of 1.08% to 7.46%, which was fully contained within the predefined equivalence margin of ±15.1%. Together, these findings indicate that fUS can repeatedly detect whisker stimulus‐evoked functional hyperemia in awake mice over multiple months, with reproducible spatial localization across sessions and no evidence of systematic drift in the mean response over time.

**TABLE 1:** Longitudinal variability, reproducibility, and detection thresholds of multimodal ultrasound metrics.

Domain	Metric	Pooled within‐subject CV (%)	ICC(2,1)	RC	Linear trend P	Empirical range	MDC95
Structural	Vascularity (%)	7.7	0.48	16.1	0.901	60.2–91.5	16.1
Flow	Mean flow velocity (mm/s)	6.4	0.72	1.4	0.866	6.3–9.9	1.4
Functional	fUS ΔCBV (%)	15.4	0.47	15.1	0.306	19.2–51.2	15.1
	fULM ΔMB flow (%)	28.9	0.60	11.5	0.973	0.4–28.1	11.5

*Abbreviations*: CV, coefficient of variation; ICC, intraclass correlation coefficient; MDC95, minimal detectable change at the 95% confidence level. The pooled within‐subject CV was calculated as SDwithin divided by the pooled longitudinal mean and multiplied by 100%, where SDwithin was estimated as the square root of the residual mean square from a two‐way mouse‐by‐month ANOVA. ICC was estimated using a two‐way random‐effects, absolute‐agreement, single‐measure model, ICC(2,1). RC was calculated as 1.96 × √2 × SDwithin. MDC95 was calculated as 1.96 × √2 × SEM, where SEM was estimated as the square root of the same ANOVA residual mean square. Therefore, RC and MDC95 were numerically equivalent in the present analysis. Linear trend *p* values were obtained from linear mixed‐effects models with month entered as a continuous fixed effect and mouse identity included as a random intercept. Study‐specific empirical ranges were calculated as the pooled longitudinal mean ± 2 pooled SD and should be interpreted as descriptive ranges for the present cohort rather than population‐level reference intervals; RC, repeatability coefficient; SEM, standard error of measurement.

### 2.2 ULM Indicates Stable Microvascular Architecture and Flow Velocity

**FIGURE 3:**
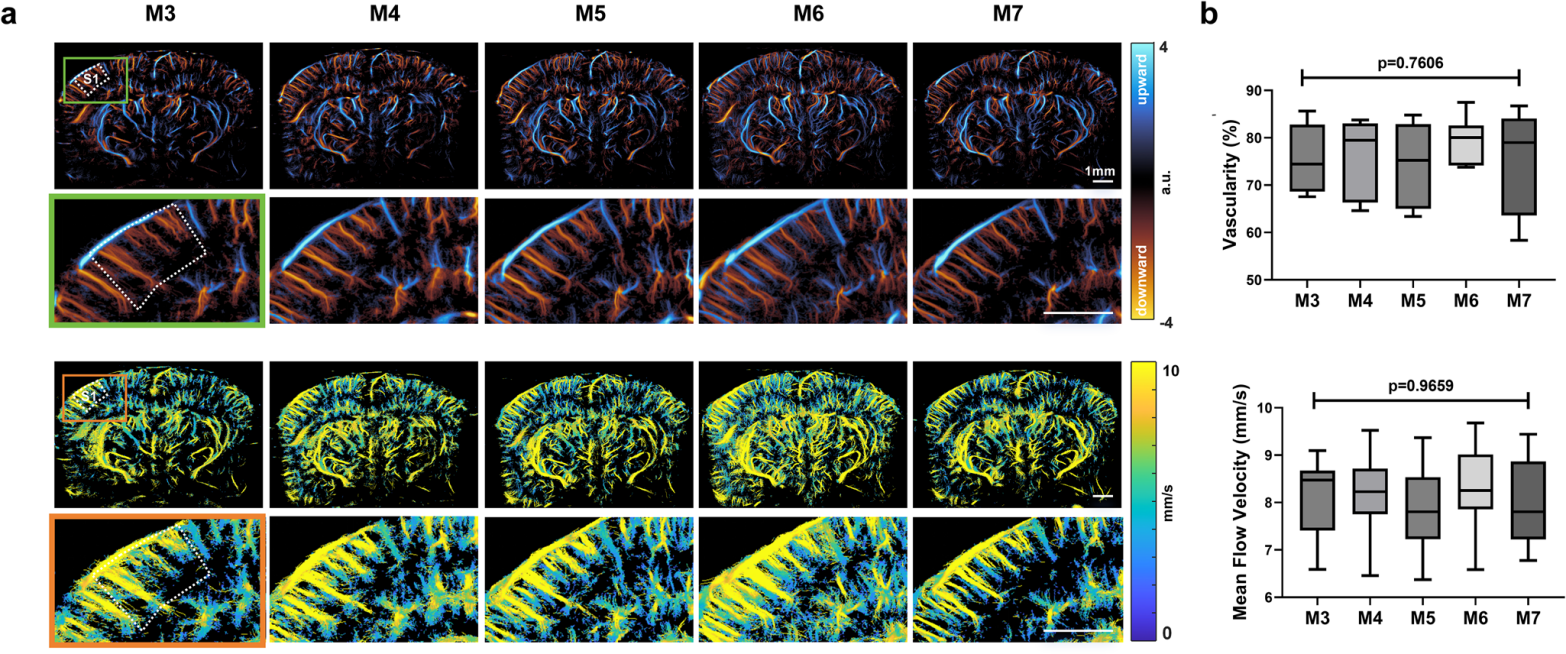
Longitudinal ULM imaging reveals stable microvascular structure and flow velocity. (a) Directional flow maps specifically within the S1 region illustrating arterial (red, downward flow) and venous (blue, upward flow) components from 3 to 7 months. Flow velocity maps acquired from 3 to 7 months. (b) Group‐level analysis of vascularity and flow velocity within the S1 region (*n* = 7). Data are presented as box‐and‐whisker plots showing the median and IQR. ULM, ultrasound localization microscopy; S1, primary somatosensory cortex; IQR, interquartile range.

Figure 3 presents the ULM imaging results across five imaging sessions throughout 5 months. Within the S1 cortex (white dotted ROI in Figure 3a), vascularity remained stable across all time points, with no significant month‐to‐month differences observed at the group level (one‐way ANOVA, *p* = 0.7607; Figure 3b). Vascularity values remained within a narrow range (74.0%–79.1%) across sessions, with an overall CV of 10.0%, indicating stable microvascular structural organization over time. Mean cerebral microvascular flow velocity within the S1 cortex also remained stable over time (one‐way ANOVA, *p* = 0.9659; Figure 3b). The velocity fluctuated within a narrow range (7.9–8.2 mm/s; overall CV = 11.1%). Consistent with these group‐level observations, longitudinal modeling further supported the stability of both ULM‐derived metrics. Linear mixed‐effects analysis showed no significant longitudinal trend for either mean flow velocity or vascularity (trend *p* = 0.866 and 0.901, respectively). Mean flow velocity and vascularity showed pooled within‐subject CVs of 6.4% and 7.7%, respectively, with corresponding ICC values of 0.72 and 0.48 (Table 1). Paired TOST analysis further supported equivalence between Month 3 and Month 7 for both metrics, with 90% confidence intervals fully contained within the predefined MDC_95_‐based margins (mean flow velocity: mean difference, −0.078 mm/s; 90% confidence interval, −0.849 to 0.694 mm/s; equivalence margin, ±1.4 mm/s; vascularity: mean difference, −1.97%; 90% confidence interval, −8.53% to 4.59%; equivalence margin, ±16.1%). Across ULM‐derived measures, variance decomposition indicated that within‐subject variability accounted for a minority of total variance, with inter‐animal differences predominating. Some residual variation may reflect vessel blurring caused by residual tissue motion during awake imaging.

### 2.3 fULM Indicates Stable Microvessel‐Level Hemodynamic Responses to Whisker Stimulation

**FIGURE 4:**
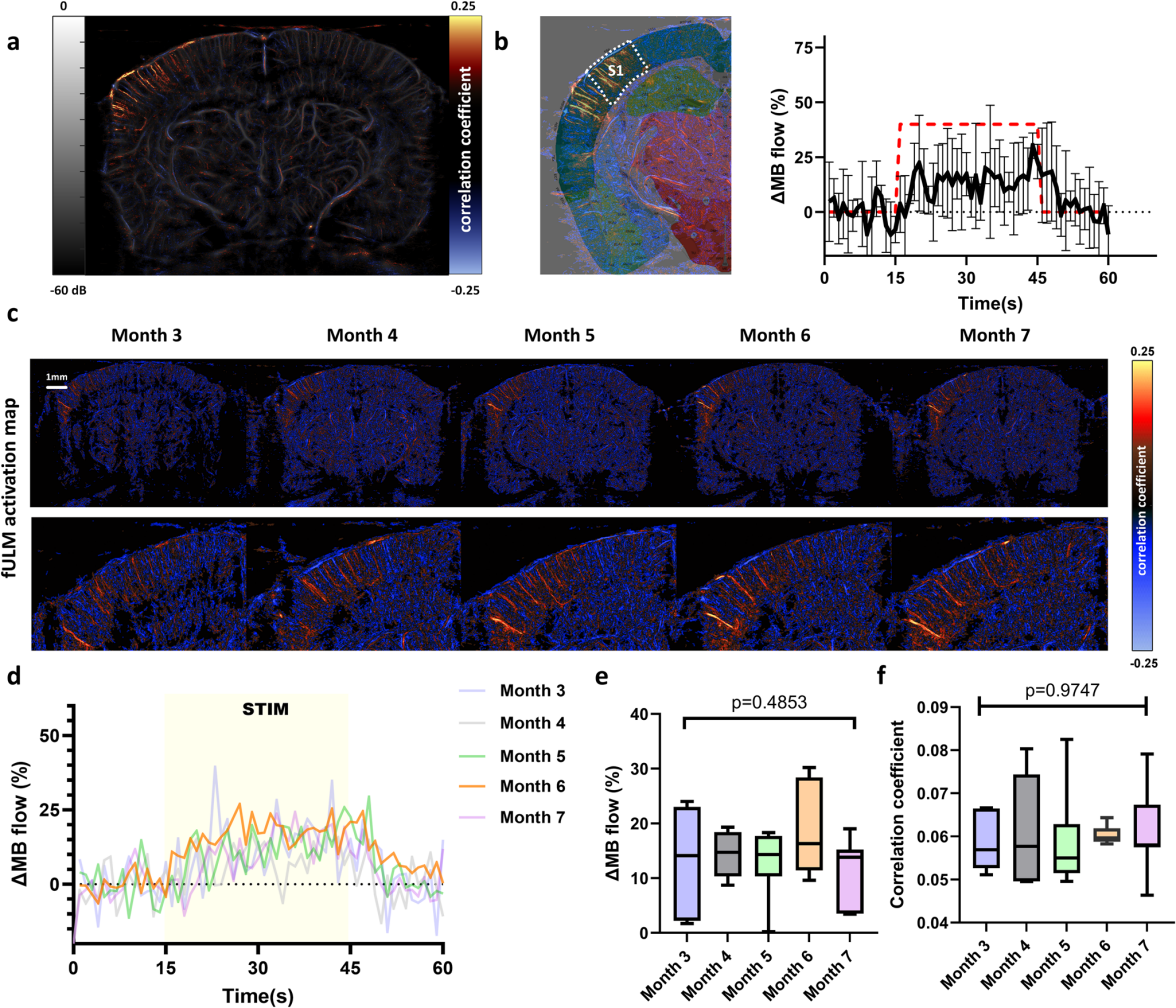
Longitudinal fULM imaging and microvessel‐level stimulus‐evoked hemodynamic responses. Representative fULM activation maps during right‐whisker stimulation, showing localized responses in the contralateral S1 cortex. (b) Averaged ΔMB flow (%) time course within the S1 region from the same animal as in (a), derived from five stimulation cycles (red dashed lines indicate the 15–45 s stimulation period; data are mean ± SEM). (c) Longitudinal fULM activation maps from the same mouse illustrating consistent microvessel‐level activation across months. (d) Averaged ΔMB flow (%) time course within the S1 region derived from five stimulation cycles of 5 months. (e, f) Group‐level analyses (*n* = 7) showing stable mean ΔMB flow (%) and flow‐stimulus correlation coefficients across five months. Data are presented as box‐and‐whisker plots showing the median and IQR. fUS, functional ultrasound; fULM, functional ultrasound localization microscopy; ΔCBV, change in cerebral blood volume.

Figure 4 shows the representative activation maps generated by fULM which reveal spatially localized microvascular responses. Within the S1 region, ΔMB flow (%) time courses exhibited synchronized blood flow increases within the stimulation window (15–45 s; Figure 4a–c,e). Because fULM measures blood flow fluctuations based on microbubble counts, which is different from fUS, the ΔMB flow (%) measurements range from 11.8% to 18.4% with an overall CV of 63.9% across all animals and sessions, indicating greater variability than fUS. fULM analysis across all five monthly sessions (3–7 months) demonstrated no significant longitudinal changes in mean ΔMB flow (%) responses (one‐way ANOVA, *p* = 0.4853; Figure 4e). Similarly, the activation measurements derived from correlation between microbubble fluctuation and the stimulus pattern remained stable across longitudinal sessions (one‐way ANOVA, *p* = 0.9747; Figure 4f). Mean ΔMB flow (%) also showed no significant longitudinal trend by linear mixed‐effects analysis (trend *p* = 0.973), but exhibited the greatest pooled within‐subject variability among all readouts, with a pooled within‐subject CV of 28.9% and an ICC of 0.60 (Table 1). Paired TOST analysis nevertheless supported equivalence between Month 3 and Month 7, with a mean difference of −2.33% and a 90% confidence interval of −6.68% to 2.03%, which was fully contained within the predefined equivalence margin of ±11.5%.

### 2.4 Longitudinal Baseline Variability, Reproducibility, and Detection Thresholds

Table 1 summarizes the longitudinal baseline variability and reproducibility statistics of multimodal ultrasound measurements acquired over the five monthly awake imaging sessions. No metric showed significant longitudinal trends, with all *p* values ≥ 0.306, which indicates the absence of systematic time‐dependent differences (Table 1). Month‐to‐month variability was lower for structural and flow metrics than for functional responses, with pooled within‐subject CVs of 6.4% for mean flow velocity, 7.7% for vascularity, compared with 15.4% for fUS‐∆CBV and 28.9% for fULM‐ΔMB flow (%) (Table 1). Mean flow velocity showed the strongest between‐animal consistency relative to within‐subject variation (ICC = 0.72) (Table 1).

Table 1 also summarizes the detection thresholds for each quantitative metric, providing practical reference values for future studies designed to detect biological differences using the fUS, ULM, or fULM. Mean flow velocity showed the lowest detection threshold, requiring a change of 1.4 mm/s to exceed baseline variability. Vascularity required a change of 16.1% to exceed baseline variability. Functional readouts required changes of 15.1% for fUS‐∆CBV and 11.5% for fULM‐ΔMB flow (%) for detectable differences. Together, these results indicate that mean flow velocity was the most consistent longitudinal readout, whereas functional responses showed higher relative variability across sessions.

### 2.5 Quantification of Functional Variability and Technical Noise Sources

Because functional readouts showed higher longitudinal variability than structural and flow‐derived ULM metrics, we further decomposed this variability into within‐session trial‐to‐trial and between‐session longitudinal components for fULM‐derived ΔMB flow. Trial‐to‐trial variability was calculated from the five repeated whisker‐stimulation trials within each imaging session, and session‐to‐session variability was estimated from monthly session‐level means using the pooled within‐subject variance framework. The pooled trial‐to‐trial variance was 6.18, corresponding to a trial‐to‐trial SD of 2.49% and a trial‐to‐trial CV of 17.4%, whereas the session‐to‐session variance was 17.07, corresponding to a within‐subject SD of 4.13% and a pooled within‐subject CV of 28.9%. Thus, the higher variability of fULM‐derived ΔMB flow was driven predominantly by between‐session factors rather than by instability across repeated stimulation trials within a session.

To further examine whether arousal state contributed to the variability of fULM ΔMB flow, we quantified pupil size and forelimb movement fraction from behavioral recordings across imaging sessions. ΔMB flow was significantly correlated with pupil size, measured as the pupil‐to‐eye area ratio (Pearson *r* = 0.757, *p* = 0.018; Spearman ρ = 0.800, *p* = 0.010, Figures S2a and S2b). Forelimb movement fraction showed a moderate but non‐significant positive association with ΔMB flow (Pearson *r* = 0.447, *p* = 0.228; Spearman ρ = 0.582, *p* = 0.100). These findings suggest that arousal‐related physiological state contributes to variability in fULM responses.

We next quantified the major technical noise sources that could affect fUS, ULM, and fULM readouts. For fUS, flashing artifacts (transient frame‐wise intensity spikes) were detected and excluded before calculating stimulus‐evoked ΔCBV. Recalculating ΔCBV with and without artifact exclusion yielded a mean absolute bias of 3.5 ± 1.4%, smaller than the ΔCBV MDC95/RC threshold of 15.1%. Residual tissue motion after correction was quantified from the remaining frame‐to‐frame displacement, which was 0.020 ± 0.008 mm. The association between residual displacement and vascularity was not significant (*p* = 0.448), indicating that residual motion contributed little measurable variance to the vascularity readout after correction.

### 2.6 Chronic PMP Cranial Window Maintains Long‐Term Cortical Biocompatibility

To investigate whether the long‐term cranial window implantation induced chronic inflammation to the brain, we performed immunofluorescence staining for GFAP (astrocytes) and Iba1 (microglia) after the five‐month imaging protocol (Figure S3a). Quantitative analysis demonstrated that GFAP and Iba1 expression levels beneath the PMP window were comparable to those in the non‐window‐covered cortex, indicating no significant glial activation (Figure S3b). Consistent with this observation, we found no morphological features of reactive astrogliosis or microglial aggregation, which are hallmarks of chronic neuroinflammation or foreign body responses. Fluorescence intensity profiles for both markers were consistent across animals, and no focal accumulation of GFAP or Iba1 was detected beneath the window. These results indicate that the surgical implantation of the PMP window, together with repeated head fixation and longitudinal imaging, does not induce sustained cortical inflammation or scarring. To evaluate whether cognitive function was affected after 5 months of repeated imaging, we assessed behavior using a passive avoidance task. Mice exhibited prolonged step‐through latencies during the retention test (Figure S3c), consistent with preserved avoidance memory.

### 2.7 Pathological Validation in 5xFAD Mice Reveals a Temporal Sequence of Cross‐Scale Microvascular Dysfunction

**FIGURE 5:**
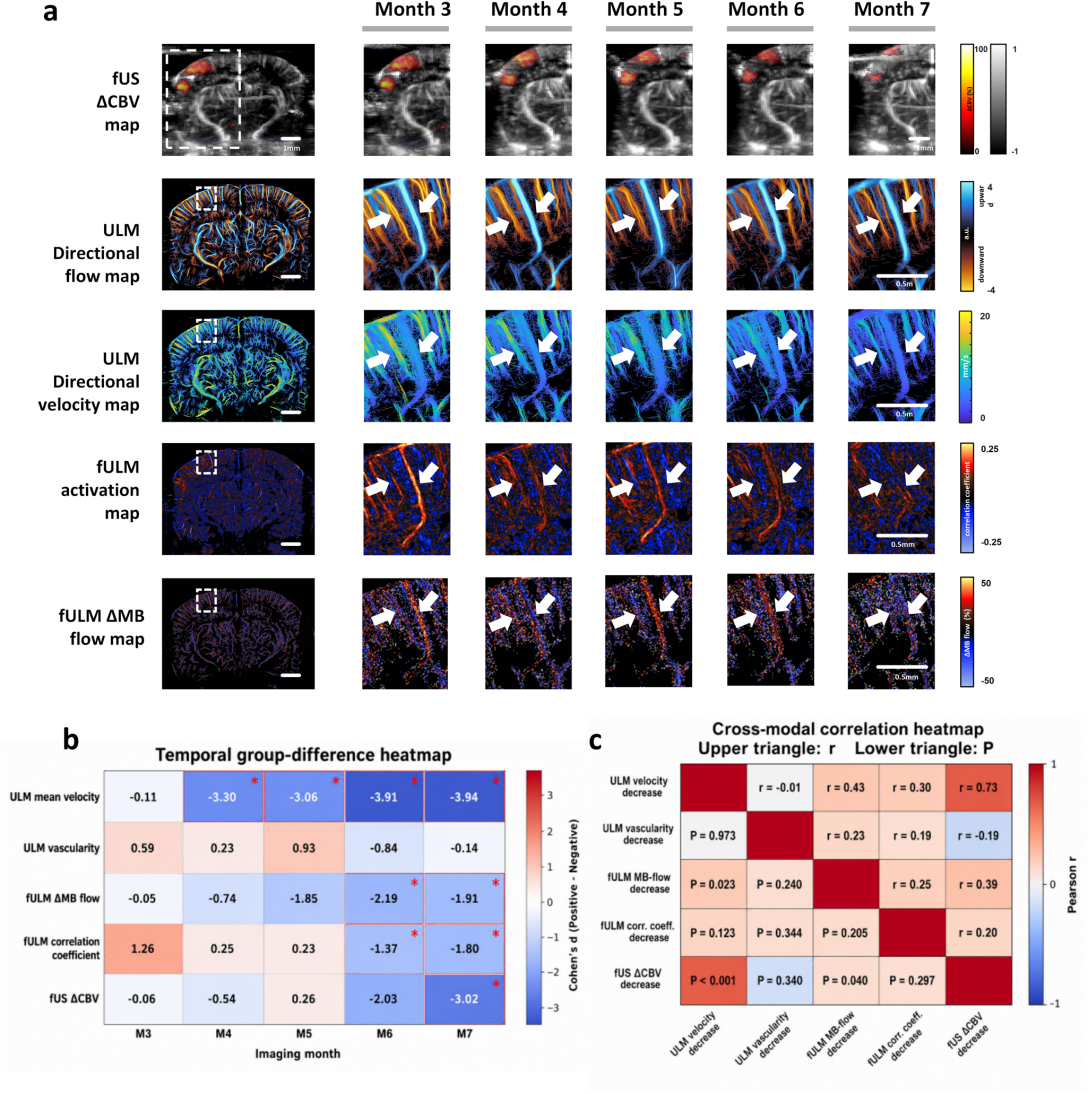
Longitudinal fUS‐ULM‐fULM imaging reveals progressive vascular structural and functional alterations in 5xFAD‐positive mice. (a) Representative longitudinal fUS ΔCBV maps, ULM directional‐flow and velocity maps, and fULM correlation‐coefficient and ΔMB‐flow maps acquired in the same imaging plane from Month 3 to Month 7 on the same 5xFAD‐positive animal. Dashed boxes in the global images indicate the regions enlarged in the subsequent longitudinal imaging panels, and white arrows highlight representative vessels that demonstrate structural and functional deteriorations over time. (b) Cross‐modal associations among longitudinal changes from baseline in the 5xFAD‐positive group. Greater decrease in vessel flow velocity is associated with greater decreases in vascular hemodynamic responses to whisker stimulation at both the microvascular and regional levels. The upper triangle shows the pooled Pearson correlation coefficients (r), the lower triangle shows the corresponding two‐sided P values. (c) Temporal heatmap of intergroup differences in ULM‐, fULM‐, and fUS‐derived measurements at Months 3–7. Red outlines and asterisks denote significant month‐specific differences between the 5xFAD‐positive and 5xFAD‐negative groups (*n* = 7 mice per group).

To determine whether the integrated awake fUS‐ULM‐fULM framework provides biological insight beyond baseline stability assessment, we extended the study to a pathological validation cohort of seven 5xFAD‐positive mice and compared them with seven negative controls using the same longitudinal awake imaging pipeline (Figure 5). The mean body weight of the 5xFAD‐positive mice was 24.4 ±  2.7 g, with no significant difference between the two groups (*p* = 0.645). Linear mixed‐effects modeling revealed significant genotype‐by‐month interactions (i.e., between 5xFAD‐positive and negative groups and across time) for mean flow velocity (*p* < 0.001), ∆MB flow (*p* = 0.0009), correlation coefficient (*p* < 0.001), and ∆CBV (*p* = 0.0102), whereas vascularity showed no significant interaction (*p* = 0.1563). As shown in Figure 5b, mean flow velocity was the earliest altered readout, with 5xFAD‐positive mice showing significantly lower velocity (5.87 ±  0.10 mm/s) than negative controls (8.17 ±  0.36 mm/s) at M4 (Sidak‐adjusted *p* < 0.001, Figure S4a). This difference remained significant at later time points, indicating continued deterioration of cerebral blood flow as AD progressed. In addition, at the individual‐animal level, all seven 5xFAD‐positive mice showed reductions in flow velocity across M4 through M7 that exceeded the baseline MDC95/RC threshold of 1.4 mm/s (Table 1), which was derived from negative control animals. In contrast, structural vascularity measurements did not show a significant genotype‐dependent difference at any month after correction (Figure S4a, *p* = 0.1563). ∆MB flow and mean stimulus correlation, which were derived from fULM that are indicative of microvascular functional responses to whisker stimulation, showed a significant deterioration in 5xFAD‐positive animals from M6 onward (Figure S4b; *p* = 0.009 and *p* < 0.001, respectively). Although fUS‐derived ΔCBV became significantly lower in 5xFAD‐positive mice than in negative controls at M7 (Figure S4c; 19.01 ± 2.26 vs. 37.00 ± 2.24; Sidak‐adjusted *p* = 0.0021), fUS remained insensitive to the microvascular functional impairment that fULM had already revealed as early as M6, owing to the lack of contrast microbubbles that boost the sensitivity of ultrasound to microvascular flow. Overall, these results suggest that flow velocity impairment preceded functional response impairment during AD pathology progression with no detectable structural abnormalities. This structural interpretation was independently supported by terminal histopathology, which confirmed unchanged vessel density and linked amyloid‐vessel proximity to the severity of flow velocity decline (Method S2, Figure S5).

Cross‐modal analyses further examined the associations among longitudinal changes in ULM‐, fULM‐, and fUS‐derived measures (Figure 5b). Within the 5xFAD‐positive group, greater baseline‐referenced decreases in mean flow velocity were associated with greater decreases in ∆MB flow (*r* = 0.43, *p* = 0.023; r_rm_ = 0.45, *p* = 0.038, repeated‐measures correlation), whereas changes in vascularity were not (*r* = 0.23, *p* = 0.240; r_rm_ = 0.25, *p* = 0.256), suggesting that functional impairment arises from reduced flow velocity rather than loss of vascular integrity. Greater decreases in ∆MB flow were also associated with greater decreases in ∆CBV (*r* = 0.39, *p* = 0.040; r_rm_ = 0.38, *p* = 0.044), indicating that macroscale hemodynamic deficits originate from microscale dysfunction. These results highlight the value of the integrated ULM‐fULM‐fUS framework in resolving cross‐scale associations that would remain inaccessible to any single modality.

## 3 Discussion

In this study, we established an integrated multiscale ultrasound imaging framework combining fUS, ULM, and fULM for awake longitudinal mouse brain imaging. Using a chronically stable PMP cranial window, we demonstrated serial multimodal acquisition in the same animals over five months and quantified baseline variability across mesoscopic hemodynamic and microvessel‐scale structural/flow metrics, providing sensitivity benchmarks for distinguishing disease‐related deviations from baseline fluctuations.

Using fUS, we detected robust whisker‐evoked hemodynamic responses in the contralateral barrel cortex of awake mice. Activation maps were used to extract ΔCBV time courses, which showed an onset within the first few second followed by a slower rise, consistent with the expected hemodynamic response dynamics. Compared with prior reports in anesthetized mice (relative CBV changes of ∼10.5 ± 3.7% at 2 months and ∼8.7 ± 2.7% at 10 months [21]), responses in awake mice were larger (ΔCBV 30%–38% from 3–7 months), which is consistent with the known influence of anesthesia on neurovascular coupling. Our study showed that awake imaging is associated with greater longitudinal variability, and our statistical analysis showed that fUS‐ΔCBV changes need to exceed 15.1% to be detectable over baseline variability. These results provide a practical benchmark for interpreting biologically meaningful differences in future awake imaging studies using fUS. We did not observe clear ventral posteromedial nucleus activation under the present stimulation and imaging conditions. Although whisker‐evoked VPM responses have been reported in previous ultrasound studies [9], their detectability may vary depending on imaging modality, sensitivity, and the experimental configuration, particularly in the awake state. In contrast, cortical activation patterns can appear spatially broader, extending beyond the S1 barrel core into adjacent cortex, consistent with thalamocortical and intracortical propagation as well as vascular integration across shared arteriolar territories [22, 23].

ULM enables super‐resolution ultrasound imaging of the cerebral microvasculature, allowing visualization of microvessel networks beyond the resolution of conventional ultrasound [24]. In healthy awake mice, ULM‐derived metrics (vascularity and mean flow velocity) remained stable over five months, providing a quantitative baseline for longitudinal studies and for detecting deviations induced by aging or disease. To our knowledge, this work represents the first longitudinal demonstration of ULM imaging stability in awake mice. Within the S1 region, ULM flow velocity showed the strongest reproducibility and lowest longitudinal variability (pooled within‐subject CV = 6.4%), with a minimal detectable change of 1.4 mm/s, while vascularity also exhibited low variability (pooled within‐subject CV = 7.7%) and required a 16.1% change to exceed baseline variability. These findings support the longitudinal stability of ULM and demonstrate that our awake longitudinal imaging procedure enables reliable repeated structural and flow quantification over months in awake animals.

fULM extends ULM by enabling microvessel‐scale mapping of stimulus‐evoked flow dynamics [13, 25]. In the present study, fULM resolved localized microvascular responses to whisker stimulation that are not accessible with fUS imaging alone. Although fULM‐derived functional metrics showed greater pooled within‐subject CV and session‐to‐session variations than mesoscopic fUS measures, they remained reproducible at the cohort level, with no evidence of systematic longitudinal drift across repeated sessions. These findings support fULM as a reproducible tool for establishing baseline microvascular functional references and for future longitudinal studies of neurovascular coupling alterations in awake mice.

The addition of the 5xFAD cohort demonstrated the utility and the complementary nature of the the proposed multimodal ultrasound imaging. Our results indicate that alterations in mean flow velocity preceded detectable changes in all other parameters including vascular functional responses and structural changes, which suggests that flow impairment may drive the structural and functional vascular damage that manifests at later stages of AD progression. Among the functional readouts, fULM‐derived ΔMB flow was the earliest at detecting significant reduction in stimulus‐evoked response, which was linked to the reduction in blood flow velocity instead of vascularity. This finding suggests that functional impairment in cerebral blood vessels associated with AD is caused by an impeded blood flow instead of structural integrity of the blood vessels. Our results also suggest that neurovascular dysfunction originates at the microvascular level (Figure 5b and Figure S4b, fULM) before manifesting at the macrovascular level (Figure 5c and Figure S4c, fUS), and that the injection of contrast microbubbles in fULM greatly enhanced the capability to detect such microvascular abnormalities at an earlier stage of disease. Taken together, none of these conclusions could have been reached without the concurrent use of all three imaging modalities in the same animals within a longitudinal study design. These findings underscore the practical utility and significance of our proposed multimodal imaging approach for studying cerebrovascular disease progression, not only in AD but in any other neurological disease or disorder involving CBF or vascular abnormalities.

Our study has several limitations. First, the head holder and residual skull bone introduced intermittent “flashing” artifacts in fUS images that may bias the ΔCBV estimates. We implemented temporal filtering and intensity thresholding to reduce these artifacts, but future studies may be needed to investigate and find solutions to systematically remove these flashing artifacts. Second, ULM can be influenced by variabilities in microbubble injection and physiological conditions of the animal. In this study we used fixed total number of microbubble counts to retrospectively control these variations but a future study may be needed to develop a prospective and adaptive microbubble concentration control method to acquire ULM data with consistent microbubble distributions in the blood stream. Third, the relatively large injection volume required for long‐duration microbubble imaging may itself influence the physiological state of awake mice, although no adverse effects were observed under the standard protocol used in this study. To explore whether the injection volume could be reduced, we performed an additional comparison using double microbubble concentration with half the pump rate. This protocol produced largely comparable ULM vascularity, ULM mean velocity, and fULM ΔMB flow compared with the standard microbubble concentration and pump rate, suggesting that future studies may reduce injection volume by increasing microbubble concentration while maintaining comparable vascular and functional readouts (Figure S6a,b). Fourth, although head fixation minimized large tissue displacements in our study, small, micron‐level movements were still present in our dataset as manifested by the blurring artifacts in the ULM images. Fifth, the modest sample size limits the precision and generalizability of the empirical ranges and detectable‐change thresholds, which require validation in larger independent cohorts. Lastly, although awake imaging preserves more physiological neurovascular dynamics than anesthesia, functional responses acquired in the awake state may still be influenced by state‐dependent factors. In the present study, stimulation timing, actuator settings, dowel position, and orientation were standardized across sessions; however, the actual whisker displacement amplitude was not directly measured. Small session‐to‐session differences in whisker engagement or deflection amplitude may therefore have contributed to variability in the evoked fUS and fULM responses. In addition, whisker‐evoked responses may be modulated by stress, arousal, or anxiety, particularly during early habituation phases, although these behavioral‐state variables were not continuously quantified in the present dataset. Future studies incorporating direct whisker‐displacement calibration, synchronized whisking monitoring, and refined awake imaging paradigms, including freely moving approaches [26] or roller‐supported head‐fixation setups [27], may help separate behavioral‐state effects from biological neurovascular variability while preserving physiological brain states.

In summary, we developed a multiscale, multimodal ultrasound imaging framework integrating fUS with ULM and fULM in awake mice, and validated its longitudinal feasibility, safety, and reliability over five months without detectable chronic neuroinflammation or memory impairment. By defining study‐specific empirical ranges and MDC95/RC‐based detection thresholds in negative mice, the framework established a quantitative basis for distinguishing disease‐related alterations from normal variability. Application to 5xFAD mice revealed that cerebrovascular dysfunction follows a temporally ordered, cross‐scale trajectory, with flow velocity declining first, followed by impaired microscale and then mesoscale functional responses, linked by significant cross‐modal correlations. Together, these findings support the longitudinal feasibility, safety, and biological utility of integrated fUS–ULM–fULM imaging and demonstrate its value for resolving cross‐scale neurovascular dysfunction in preclinical disease models.

## 4 Methods

### 4.1 Animal Preparation

Male 5xFAD‐positive mice and non‐transgenic littermate controls were included in this study (*n* = 7 per group). Male non‐transgenic littermate controls were used in this study as healthy controls. These mice were 12 weeks old at the time of surgery. Male mice were used to reduce potential biological variability associated with sex‐dependent vascular and physiological fluctuations, including those related to the estrus cycle [18]. The 5xFAD colony was maintained by crossing B6SJL‐Tg(APPSwFlLon, PSEN1M146LL286V)6799Vas/Mmjax transgenic mice with B6SJLF1/J mice. Genotyping was performed using the Transnetyx service. Mice positive for the 5xFAD transgene were assigned to the 5xFAD‐positive group, whereas genotype‐negative offspring were used as non‐transgenic controls.

All procedures were approved by the Institutional Animal Care and Use Committee of the University of Illinois Urbana–Champaign (Protocol #22033). Mice were maintained under a 12‐h light/dark cycle with ad libitum access to food and water. Animals were group‐housed before surgery and singly housed after cranial window implantation to facilitate postoperative recovery and reduce the risk of injury.

### 4.2 Pre‐Surgery Handling

Beginning one week before cranial window surgery, mice were habituated by tunnel handling, a method shown to lower anxiety in mice [28]. For the first 3 days, a commercially available polycarbonate transfer tube (TRANS‐TUBE, 130 × 50 mm; Braintree Scientific, Inc., Braintree, MA, USA) was used. Mice were encouraged to voluntarily enter the tube from the home cage, and the tube was then gently raised while the animal stayed inside for approximately 30 s. The mouse was then returned to the cage for one minute of free movement before the handling procedure was repeated. This routine was carried out twice daily. After approximately three days, the mice became accustomed to the tunnel handling process. At this point, a 3D‐printed body tube was attached to one end of the commercial tunnel, and the mice were allowed to enter the body tube voluntarily. For the subsequent five days, the body tube replaced the commercial tunnel for handling, ensuring that the mice were comfortable and familiar with the procedure.

### 4.3 Cranial Window Surgery

Cranial window implantation was performed as previously described [29, 14, 19]. To reduce perioperative brain swelling, mice received dexamethasone (0.5 mg/kg, intraperitoneal) before surgery. Anesthesia was induced with 3% isoflurane and maintained at 1.0%–1.5% throughout the procedure. After fixation in a stereotaxic frame, a midline scalp incision was made and the temporalis muscle was retracted from the skull. A 5 mm (AP) × 8 mm (ML) cranial window was opened with a dental drill under continuous sterile saline irrigation, and the skull flap was removed while preserving the dura. A polymethylpentene (PMP) sheet (Goodfellow, ME311051) was placed over the window and secured with tissue adhesive and dental cement. A headpost was attached to the remaining skull using the same materials (Super‐Bond C&B, Sun Medical Co., Ltd.). The window was then sealed with biocompatible silicone rubber (Body Double‐Fast Set, Smooth‐On). After the silicone cured, anesthesia was discontinued and the animal was allowed to recover.

### 4.4 Post‐Surgical Care and Head‐Fix Habituation

Postoperative analgesia was provided by subcutaneous carprofen (5–10 mg/kg) immediately after surgery. Mice were placed in individual cages post‐surgery and monitored every 15 min until regaining sternal recumbency. If signs of discomfort were observed, additional doses of Carprofen (5–10 mg/kg) were administered subcutaneously every 24 h, as needed, for up to five days. Animals were monitored daily for two weeks to ensure proper healing. Following recovery, mice underwent daily head‐fixation habituation, beginning with voluntary entry into a 3D‐printed body tube. Head‐fixation duration was progressively increased from 10 min on day 1 to 1 h by day [14]. Sessions were immediately stopped if animals exhibited signs of distress, including excessive struggling or vocalization.

### 4.5 Experimental Procedure of Imaging Sessions

On each imaging day, mice were guided into the body tube, which was fixed to the imaging table, and head fixation was achieved by securing the headpost to the tube. The headpost was chronically fixed to the skull and remained stable relative to the skull within each animal throughout the longitudinal study. The protective silicone layer was removed from the cranial window using forceps, after which ultrasound coupling gel was applied and the transducer was positioned to identify the imaging plane. During the first session, the target coronal plane was defined at bregma −1.5 mm using anatomical landmarks. Once identified, the relative position of the probe and headpost was recorded to ensure consistent repositioning in subsequent sessions. B‐mode and power Doppler images were also saved to assist relocation of the same imaging plane over time. Because minor differences in headpost placement could occur between animals, the imaging plane at each session was confirmed using anatomical and major vascular landmarks in the B‐mode and power Doppler images, rather than relying on a fixed probe‐headpost mechanical position.

Imaging was performed using a Vantage 256 ultrasound system (Verasonics Inc., Kirkland, WA) equipped with an L22–14vX linear‐array transducer (Verasonics Inc.). The probe was mounted in a custom 3D‐printed holder attached to a motorized translation stage (VT‐80, Physik Instrumente, Auburn, MA), enabling precise adjustment in the elevational direction to target the desired coronal plane. For each mouse, imaging was performed in a fixed sequence of task‐evoked fUS, ULM, and task‐evoked fULM. Following fUS, an interval of approximately 15 min was required for brief anesthesia, tail‐vein catheter placement, and recovery to the awake state. ULM and fULM acquisitions were subsequently performed consecutively.

#### 4.5.1 Task‐Evoked fUS

To minimize motion artifacts, mice were head‐fixed and acclimated for at least 5 min before data collection. Imaging was performed in an awake state under low‐light conditions to reduce external stimuli that might interfere with spontaneous neural activity. Whisker stimulation was delivered using a custom‐built device, an Arduino‐controlled wooden dowel oscillated at a rate of 3 Hz within a fixed angular range, to deflect the mouse's whiskers. At each session, the wooden dowel was positioned approximately 5–7 mm from the targeted mystacial whisker pad and oriented perpendicular to the whiskers to produce whisker deflection without direct skin contact. Whisker stimulation was synchronized with ultrasound acquisition. At the start of each stimulation acquisition, the Verasonics system sent an output trigger to the Arduino, which initiated the predefined whisker‐stimulation sequence by driving the wooden dowel. Each trial lasted a total of 360 s, including 30 s of baseline recording, 5 on‐off blocks (30 s of stimulation, 30 s of rest in each block), and 30 s of post‐stimulation rest. Ultrasound was transmitted at a center frequency of 15.625 MHz with a 5‐angle compounding sequence (−2°, −1°, 0°, 1°, and 2°) that results in a post‐compounding frame rate of 500 Hz. Each acquisition used a Doppler ensemble size of 250 frames, which were beamformed in real‐time using the Verasonics reconstruction program. The final fUS imaging frame rate was established at 1 Hz for functional imaging.

#### 4.5.2 ULM

Prior to tail vein cannulation, mice were briefly anesthetized with isoflurane (3% induction, 1% maintenance) to minimize pain and stress. A 29‐gauge catheter was inserted into the tail vein of each mouse. The needle hub and proximal catheter segment were secured to the tail using narrow strips of adhesive bandage, while sufficient slack was left in the remaining tubing to allow tail movement without applying tension to the catheter insertion site. Anesthesia was subsequently discontinued, and the animals were allowed to recover and regain full movement of their limbs and tail. A programmable infusion pump (NE‐300, New Era Pump Systems Inc., Farmingdale, NY) was used to administer fresh, activated Lumason (Bracco Diagnostics Inc., Monroe Township, NJ, USA) at a rate of 30 µl/min through the tail vein catheter. Additional information regarding the concentration, size distribution, pharmacokinetic characteristics, and representative morphology of Lumason microbubbles is provided in Method S3 and Figure S7. For each imaging experiment, a new vial of Lumason was freshly reconstituted with 5 mL of sterile 0.9% (w/v) sodium chloride solution according to the manufacturer's instructions, with no additional dilution before infusion. During imaging, a ceramic magnetic stir bar placed inside the syringe was driven by an external rotating magnet every 5 min to gently mix the MB suspension and minimize concentration changes caused by microbubble flotation. The same imaging sequence for fUS described above was used for ULM and fULM, except that fUS data were acquired at a transmit voltage of 40 V, whereas ULM and fULM data were acquired at 6 V. Instead of real‐time beamforming, for ULM, we elected to continuously acquire 800 imaging frames per second (in the format of raw, unbeamformed RF channel data) to facilitate robust ULM reconstruction. Offline beamforming was subsequently performed using the Verasonics reconstruction program after completion of the experiment.

#### 4.5.3 Task‐Evoked fULM

The procedure involved administering fresh, activated Lumason through the tail vein catheter at a rate of 60 µl/min. The whisker stimulation paradigm was identical to that used in the fUS experiments (5 on‐off blocks, 360s in total). The data collection procedure followed the ULM acquisition, which continuously saved 800 frames of RF data per second (1000 Hz PRF) for subsequent offline beamforming.

### 4.6 Processing of Images and Image Analysis

#### 4.6.1 fUS

Raw ultrasound data were processed using a spatiotemporal clutter filter based on singular value decomposition (SVD) to suppress tissue motion and background noise [29]. The first 50 singular values, primarily representing tissue‐related components, were discarded. The remaining signals were averaged over time to generate power Doppler images that served as CBV reference maps. For each session, we then derived two functional maps from the CBV time series: a *z*‐score activation map, which captures the statistical significance of stimulus evoked responses, and a ΔCBV map, which quantifies the response magnitude. The *z*‐score was calculated according to Fisher's transform with the following formula: $z_{score}=\sqrt{n-3\;}\;Arctanh\;(c)$, where n denotes the total number of time samples in the session (e.g., *n* = 360 in this case), and *c* represents the Pearson's correlation coefficient between the time series of each pixel (360 time points) and the stimulation pattern [19]. A Bonferroni‐corrected significance threshold of *p* < 0.05 was applied to identify activated regions [30]. Finally, the *z*‐score was overlaid on grayscale power Doppler images for visualization. ΔCBV was computed as a percent change relative a block‐specific pre‐stimulus baseline. Specifically, within each stimulus block, all samples from the 15‐s pre‐stimulus window, the subsequent 30‐s stimulation, and the 15‐s post‐stimulation rest were normalized to the mean CBV measured during the 15 s immediately preceding that block's stimulus onset. We refer to the concatenated 15‐s pre‐stimulus, 30‐s stimulation, and 15‐s post‐stimulation rest within each block as a “stimulation cycle.” A ΔCBV map was then generated by averaging the ΔCBV values across the 30‐s stimulation period within each cycle. For visualization of the ΔCBV maps, a correlation‐based mask was applied to suppress low‐signal regions. Specifically, only pixels with a correlation value greater than 0.3 were retained, and ΔCBV was displayed within this masked region. To enable anatomical quantification, each imaging was manually aligned to the corresponding anatomical plane of the Allen Mouse Brain Atlas based on visible structural landmarks, and regions of interest (ROIs) were delineated according to atlas‐defined boundaries. The fUS‐derived mean ΔCBV within ROI was then computed for subsequent statistical analysis.

#### 4.6.2 ULM and fULM

SVD filtering was used to extract MB signals from the tissue background in each IQ dataset. An adaptive SVD threshold was chosen to better accommodate variable tissue motion in the experimental data [31, 32]. A 2D Gaussian function was fitted to the axial and lateral dimensions of manually selected MBs to represent the system's Point Spread Function (PSF). After applying SVD filtering (Figure S8), MB separation [33] was performed on the IQ data, followed by 2D spline interpolation [34] to an isotropic resolution of 4.928 µm in both axial and lateral dimensions. The interpolated dataset underwent normalized 2D cross‐correlation with the empirical PSF to identify candidate MB locations. Pixels with low cross‐correlation coefficients were excluded [33], and regional maxima in the resulting cross‐correlation map were used to identify the centroid locations of individual MBs [35]. Tracking and trajectory estimation of MB centroids were carried out using the uTrack algorithm [36], with a minimum trajectory length of 10 frames (10 ms) applied for the super‐resolution reconstruction. To compensate for inter‐session spatial offsets during longitudinal accumulation, a post hoc rigid in‐plane motion correction based on normalized cross‐correlation was applied to ULM maps. Translational offsets in the axial and lateral directions were estimated from the microbubble density maps and used to realign all ULM images by linear interpolation before accumulation; acquisitions with estimated displacements greater than 100 pixels in either the axial or lateral direction were excluded. Because the number of excluded acquisitions could vary among datasets, the final ULM reconstruction for each imaging session was generated by accumulating a fixed total of (5 × 10^6^) accepted MB localizations. This MB‐count‐based accumulation strategy standardized the sampling density across imaging sessions, despite potential differences in the number of accepted acquisitions. The accumulated data from each acquisition session were used to generate the final ULM vascular reconstruction of the cerebral vasculature. ROIs were delineated according to atlas‐defined anatomical boundaries. Brain vascularity within each ROI was quantified from the accumulated the super‐resolution vessel map. The map was binarized using a fixed zero‐count threshold. Pixels with a nonzero accumulated MB count were classified as vessel pixels. ULM‐derived vascularity was calculated as the percentage of vessel pixels relative to the total number of pixels within the ROI. ULM‐derived flow velocity was determined for every MB track directly from the frame‐to‐frame displacement of detected MB centroids.

#### 4.6.3 Stimulus‐Evoked fULM

For fULM, the pre‐processed IQ datasets underwent the same SVD‐based clutter filtering, MB localization, and tracking procedures as described for ULM. For fULM, the exclusion criterion used for ULM accumulation was not applied to the fULM datasets because preservation of the complete temporal sequence was required for stimulation–rest analysis. All data acquired during the 360‐s functional imaging session were therefore retained. The functional response was quantified as the difference in MB count between the stimulation and rest periods within the same imaging session, calculated as stimulation minus rest. fULM‐derived ΔMB flow (%) = [(MB count_stimulation_ − MB count_rest_)/MB count_rest_] × 100%. In addition to vascular reconstruction, the temporal dynamics of MB counts were extracted at each pixel to capture hemodynamic responses. Specifically, the number of localized MBs was accumulated in one‐second frame over the duration of each trial, resulting in a MB count time series. For each pixel, the MB count trace was correlated with the stimulation pattern using Pearson's correlation analysis, yielding a correlation coefficient map. Finally, the functional correlation maps were registered to the atlas for quantitative analysis of fULM‐derived correlation coefficient within specific brain regions.

### 4.7 Histological Analysis

Brain tissue was harvested and fixed in 4% paraformaldehyde in PBS at 4°C for 24–48 h, then cryoprotected in 10%, 20%, and 30% sucrose solutions. Fixed brains were embedded and sectioned at 20 µm thickness using a cryostat (CM1850, Leica, NJ, USA). Sections were washed in TBS and permeabilized with 0.3% Triton X‐100 (Sigma‐Aldrich, St. Louis, MO, USA), then blocked with 0.3% Triton X‐100, 5% normal goat serum (Sigma‐Aldrich, St. Louis, MO, USA), and 3% BSA (Sigma‐Aldrich, St. Louis, MO, USA). Primary antibodies‐Rabbit Anti‐GFAP (Abcam, ab7260; 1:1000) and Goat Anti‐Iba1 (Novus, NB100‐1028; 1:1000)‐were applied overnight at 4°C, followed by Alexa Fluor 488‐conjugated anti‐rabbit IgG (Invitrogen, A‐11008; 1:1000) and Alexa Fluor 594‐conjugated anti‐goat IgG (Invitrogen, A‐11058; 1:1000) for 2 h at room temperature. After final washes, sections were mounted with VECTASHIELD Antifade Mounting Medium with DAPI (Vector Laboratories, Burlingame, CA, USA) and imaged using a Zeiss LSM 710 confocal microscope. GFAP, Iba1, and DAPI signals were acquired using 488 nm, 561 nm, and 358 nm excitation lasers, respectively, under identical acquisition settings. Maximum intensity projections were generated using Zen Blue. ROIs were manually defined based on DAPI morphology, and fluorescence intensities for GFAP and Iba1 were quantified in ImageJ with background subtraction.

### 4.8 Passive Avoidance Test

The passive avoidance test was conducted using a two‐compartment apparatus consisting of an illuminated chamber and a dark chamber equipped with a grid floor for foot shock delivery [37]. Mice were trained once daily from D1 to D7. During each training session, the mouse was placed in the illuminated chamber, and the step‐through latency to enter the dark chamber was recorded. Upon entry, a brief foot shock was delivered. Training latency was capped at 120 s. On D8, mice underwent the retention test, during which step‐through latency was measured again as an index of memory retention, with a maximum cutoff time of 300 s.

### 4.9 Statistical Analysis

Statistical analyses were performed using GraphPad Prism (version 9.1.2, GraphPad Software, CA, USA) and Python (version 3.9, Python Software Foundation, USA). For stimulus‐evoked hemodynamic responses measured by fUS and fULM, ΔCBV and ΔMB flow (%) time courses were averaged across five repeated stimulation trials within each imaging session to generate one session‐level mean value per animal. These session‐level summary values were used for all subsequent analyses. For longitudinal imaging analyses, *n* = 7 biologically independent mice were included, each measured repeatedly across five monthly sessions. Individual animals were treated as biological replicates, whereas the five repeated stimulation trials within each imaging session were treated as repeated measurements rather than independent replicates. To assess month‐to‐month differences in the figure panels, session‐level values for fUS ΔCBV, ULM‐derived vascularity, ULM‐derived mean flow velocity, fULM‐derived ΔMB flow, and fULM flow‐stimulus correlation were compared across the five monthly sessions using one‐way repeated‐measures analysis of variance (ANOVA). All tests were two‐sided, and a p value < 0.05 was considered statistically significant. To evaluate long‐term stability, paired two one‐sided tests (TOST) were performed to compare Month 3 and Month 7 measurements. Equivalence bounds were defined for each metric using the MDC_95_. Statistical equivalence was concluded when the 90% confidence interval of the paired difference was fully contained within the corresponding equivalence margin, with both one‐sided tests significant at α = 0.05.

To assess longitudinal stability and potential systematic time‐dependent drift while accounting for repeated measurements within animals, session‐level values from the 5xFAD‐negative control mice were analyzed using linear mixed‐effects models, with month entered as a continuous fixed effect and mouse identity included as a random intercept. The two‐sided p value for the fixed effect of month was used to test for a linear longitudinal trend [38]. Longitudinal variability and measurement reliability were further characterized using the pooled within‐subject coefficient of variation (CV), intraclass correlation coefficient (ICC), repeatability coefficient (RC), study‐specific empirical range, and minimal detectable change at the 95% confidence level (MDC_95_). The pooled within‐subject CV was calculated as: 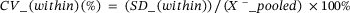. Here, 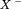 denotes the pooled longitudinal mean, and the within‐subject standard deviation was calculated as: SD_(within)=√(MS_error). The MS_error term was obtained from the residual mean square of a two‐way ANOVA with mouse identity and month as factors, without an interaction term [39, 40]. Measurement reliability was assessed using a two‐way random‐effects, absolute‐agreement, single‐measure intraclass correlation coefficient, ICC(2,1). ICC estimates were reported with their corresponding 95% confidence intervals [41]. The repeatability coefficient was calculated as: RC=1.96×√2×SD_(within) [39]. The minimal detectable change at the 95% confidence level was calculated as: MDC_95=1.96×√2×SEM, where the standard error of measurement was estimated as: SEM=√(MS_error)[42]. Because RC and MDC_95_ were derived from the same residual measurement‐error component, they were numerically equivalent in the present analysis. Study‐specific empirical ranges were calculated as: $X^{-}-pooled\pm 2SD$. These ranges were interpreted as descriptive ranges for the present cohort rather than population‐level reference intervals.

Longitudinal differences between 5xFAD‐positive mice and negative controls were analyzed using linear mixed‐effects models, with genotype, month, and the genotype‐by‐month interaction included as fixed effects and mouse identity included as a random intercept. For this analysis, month was treated as a categorical variable to allow for non‐linear longitudinal changes. Month‐specific comparisons between 5xFAD‐positive and negative mice were additionally performed using two‐way ANOVA followed by Sidak's multiple comparisons test, with adjusted p values reported for the between‐group comparison at each month. The magnitude and direction of each month‐specific intergroup difference were summarized using Cohen's d, calculated as the difference between the group means (5xFAD‐positive minus 5xFAD‐negative) divided by the pooled standard deviation. For cross‐modal analyses, Month 3 was defined as baseline, and monthly changes were calculated as the baseline value minus the corresponding follow‐up value, with positive values indicating decreases from baseline. Cross‐modal associations within the 5xFAD‐positive group were assessed using pooled Pearson correlation and repeated‐measures correlation analyses between ULM‐derived microvascular metrics, fULM‐derived ΔMB flow, and fUS‐derived ΔCBV. Results are reported as repeated‐measures correlation coefficients (r_rm_) and pooled Pearson's r, with two‐sided p values. All statistical tests were two‐sided, and p values < 0.05 were considered statistically significant.

## Author Contributions

Conceptualization and funding acquisition: P. Song, D. A. Llano; Methodology: P. Song, Z. Huang, Y. Wang, M. R. Lowerison, Y. Xu, M‐R. Yi, Y. Shin, and B.‐Z. Lin; Software: P. Song, Y. Wang and M. R. Lowerison; Validation: Z. Huang and Y. Wang; Formal Analysis: Z. Huang; Investigation: Z. Huang and Y. Wang; Resources: B.‐Z. Lin, N. V. C. Sekaran, D. A. Llano, and P. Song; Data Curation: Z. Huang; Writing – original draft: Z. Huang, Y. Wang, P. Song; Writing – review & editing: All authors; Visualization: Z. Huang, Y. Wang, Y. Xu, and Y. Shin; Project administration: P. Song. All authors have read and approved the final version of the manuscript.

## Conflicts of Interest

The authors declare no conflicts of interest.

## Supporting information




**Supporting File**: advs77931‐sup‐0001‐SuppMat.pdf.

## Data Availability

The data that support the findings of this study are available on request from the corresponding author. The data are not publicly available due to privacy or ethical restrictions.
