# Supplementary material for: Longitudinal Awake Mouse Brain Imaging Using Functional Ultrasound and Functional Ultrasound Localization Microscopy
Source: Adv Sci (Weinh). 2026 Sep 27:e77931. Online ahead of print. doi: 10.1002/advs.77931 (PMC13616189; doi:10.1002/advs.77931)
Supplement: Supplementary file 1 [file ADVS-9999-e77931-s001.pdf]

Supplementary

Supplementary Methods 1 Quantification of technical variability

Transient flashing artifacts in the fUS power Doppler time series were identified based on frame-wise intensity changes exceeding the predefined artifact threshold. Frames classified as flashing artifacts were corrected or excluded before calculation of stimulus-evoked ΔCBV. To assess the potential effect of these artifacts, ΔCBV was calculated before and after correction and compared.

The same microbubble concentration and syringe-pump infusion rate were used across imaging sessions. Because the effective number of microbubbles reaching the cerebral circulation may nevertheless vary across animals and sessions, each ULM map was accumulated using a fixed total number of accepted microbubble localizations or tracks after motion correction and quality-control exclusion. For fULM, ΔMB flow was calculated as the difference between stimulation and rest within the same acquisition.

Residual tissue displacement was recorded after translational motion correction. The relationship between residual post-correction displacement and vascularity was assessed using Pearson correlation analysis.

Supplementary Methods 2 Histological validation of imaging metrics

To determine whether imaging-detected vascular abnormalities reflected underlying AD pathology, we performed terminal histological analysis of Aβ deposition and CD31-positive vasculature. Aβ plaque area fraction was markedly

higher in 5xFAD(+) than 5xFAD(−) mice ($p = 0.007$), confirming substantial amyloid pathology at the terminal time point, whereas CD31-positive vessel area fraction did not differ between groups ($p = 0.710$), consistent with the preserved vascularity observed by longitudinal ULM imaging. Within 5xFAD(+) mice, histological vessel area fraction strongly correlated with ULM-derived vascularity (Spearman $\rho = 0.937$, $p = 0.002$), providing independent pathological validation of the imaging measurement. Within 5xFAD(+) mice, shorter median plaque-to-vessel distance was associated with a more negative longitudinal slope of ULM flow velocity (Spearman $\rho = 0.786$, $p = 0.036$).

Supplementary Methods 3 Physicochemical characteristics of Lumason® microbubbles

Lumason® consists of sulfur hexafluoride gas encapsulated by a phospholipid shell. For each imaging experiment, one vial was freshly reconstituted with 5 mL of sterile 0.9% (w/v) sodium chloride solution according to the manufacturer's instructions. The resulting suspension contained approximately $1.5\text{-}5.6 \times 10^8$ microbubbles/mL, and no additional dilution was performed before the suspension was loaded into the infusion syringe. The microbubbles have a reported mean diameter of approximately 1.5-2.5 μm, with at least 99% of the microbubbles measuring ≤10 μm [1].

The reported terminal blood half-life of the sulfur hexafluoride gas component is approximately 10 min following intravenous administration at a dose of 0.3 mL/kg in

humans [2]. This pharmacokinetic value refers to the sulfur hexafluoride gas component under the reported clinical dosing condition and was not directly measured in the mice included in the present study.

During infusion, a ceramic magnetic stir bar placed inside the syringe was driven by an external rotating magnet every 5 min to maintain a homogeneous microbubble suspension and minimize concentration changes caused by microbubble flotation. A representative bright-field microscopy image of freshly reconstituted Lumason® microbubbles is provided in Supplementary Figure 2.

1 Bracco Diagnostics Inc., LUMASON® (Sulfur Hexafluoride Lipid-Type A Microspheres) for Injectable Suspension: Full Prescribing Information, U.S. Food and Drug Administration, Silver Spring, MD 2021.
2 U.S. Food and Drug Administration, Clinical Pharmacology and Biopharmaceutics Review: Lumason (Sulfur Hexafluoride Lipid-Type A Microspheres), NDA 203684, Center for Drug Evaluation and Research, Silver Spring, MD 2014.

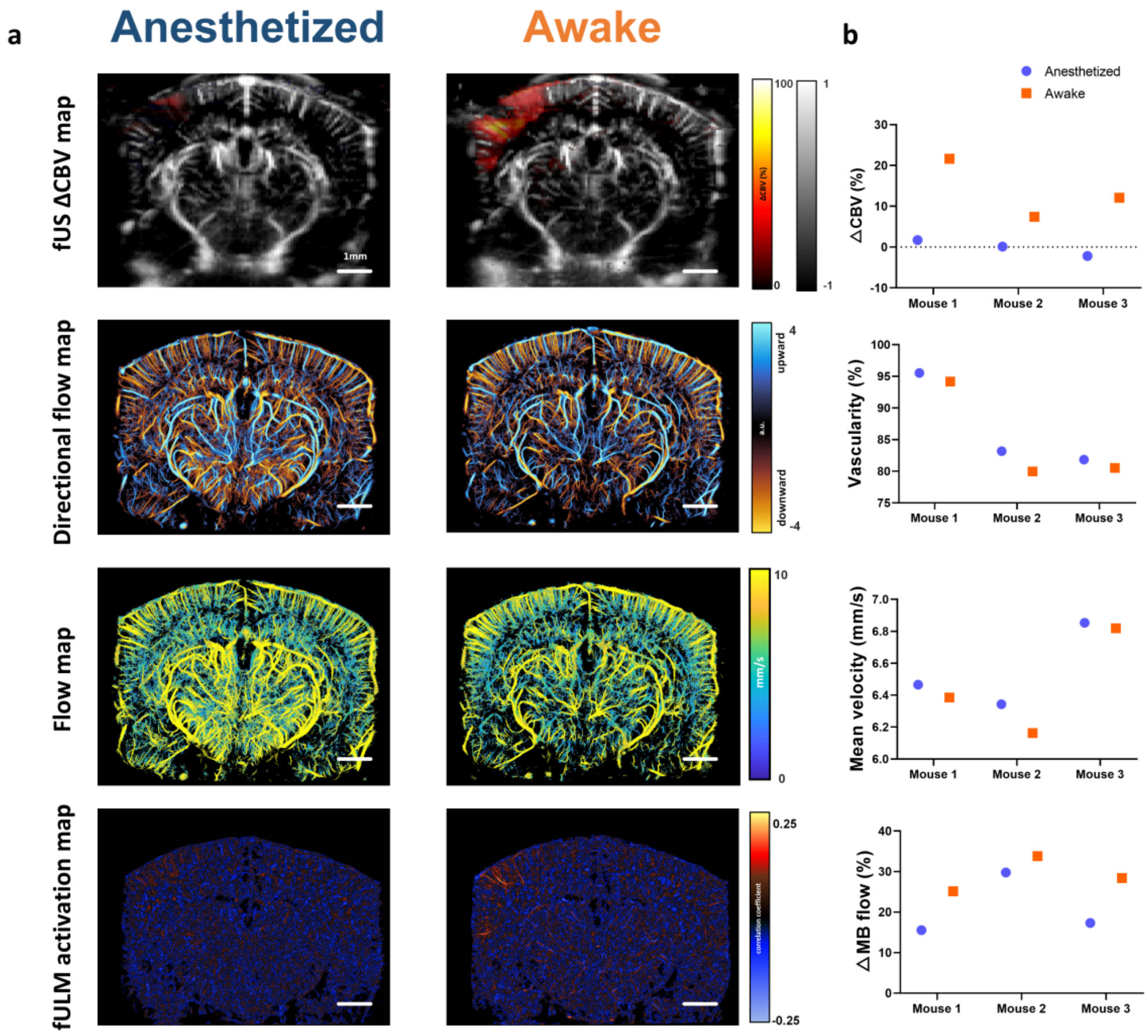

Supplementary Fig. 1. Comparison of multimodal ultrasound readouts under anesthetized and awake conditions

a, Representative multimodal ultrasound images acquired from the same animals under anesthetized and awake conditions. From top to bottom: fUS ΔCBV map, ULM directional flow map, ULM mean flow velocity map, and fULM activation map. b, Paired comparison of fUS-derived ΔCBV, ULM-derived vascularity, ULM-derived mean flow velocity, and fULM-derived ΔMB flow between anesthetized and awake conditions in the same three mice. ΔCBV and ΔMB flow were higher under awake conditions in all three animals, whereas vascularity and mean flow velocity showed smaller and less consistent differences between conditions.

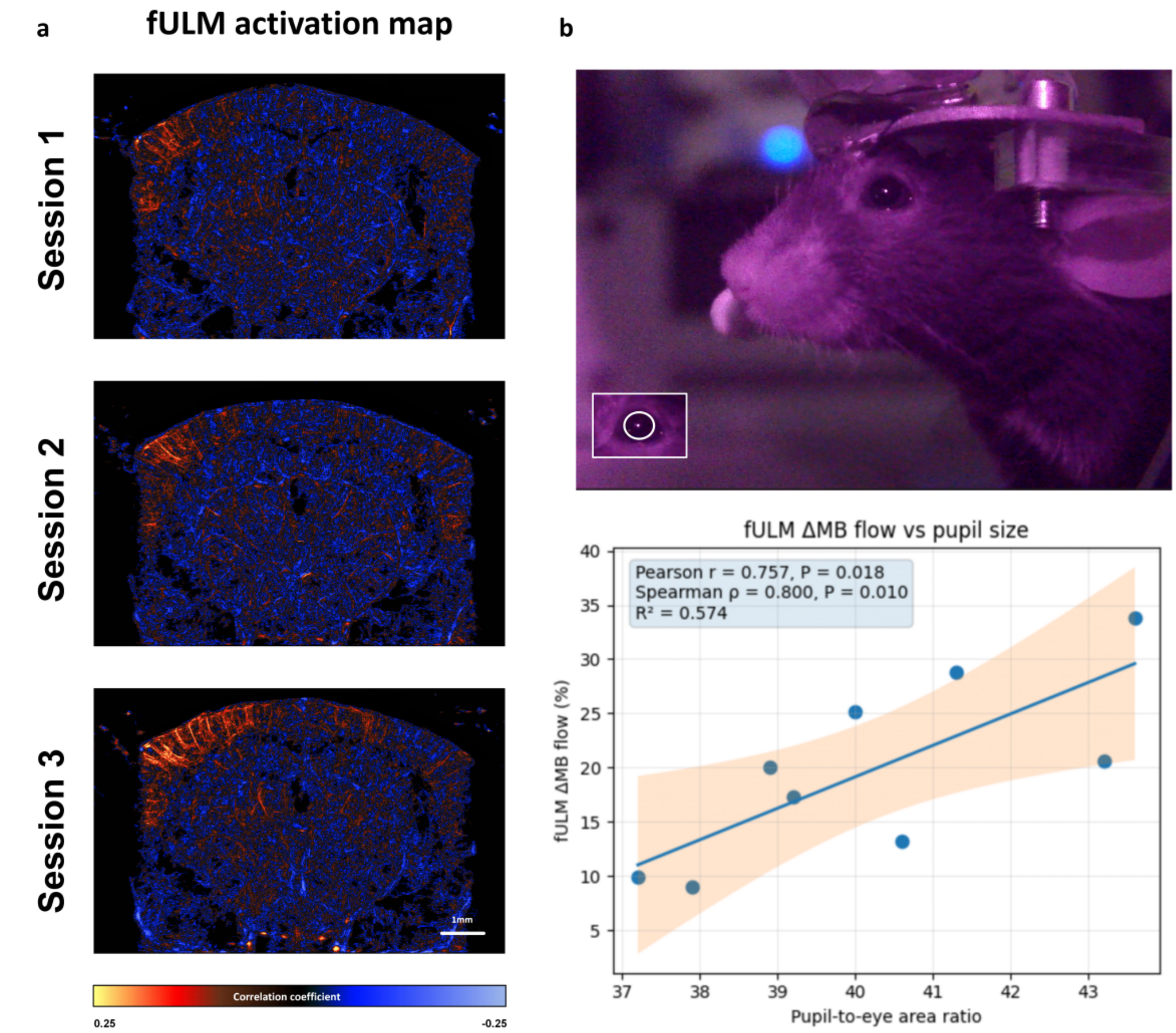

Supplementary Fig. 2. Session-dependent variability of fULM responses and association with pupil size

a, Representative fULM activation maps from three imaging sessions, illustrating session-to-session variability in MB-flow. (b) Representative pupil image acquired during awake, head-fixed imaging. fULM ΔMB flow was positively correlated with pupil-to-eye area ratio across imaging sessions.

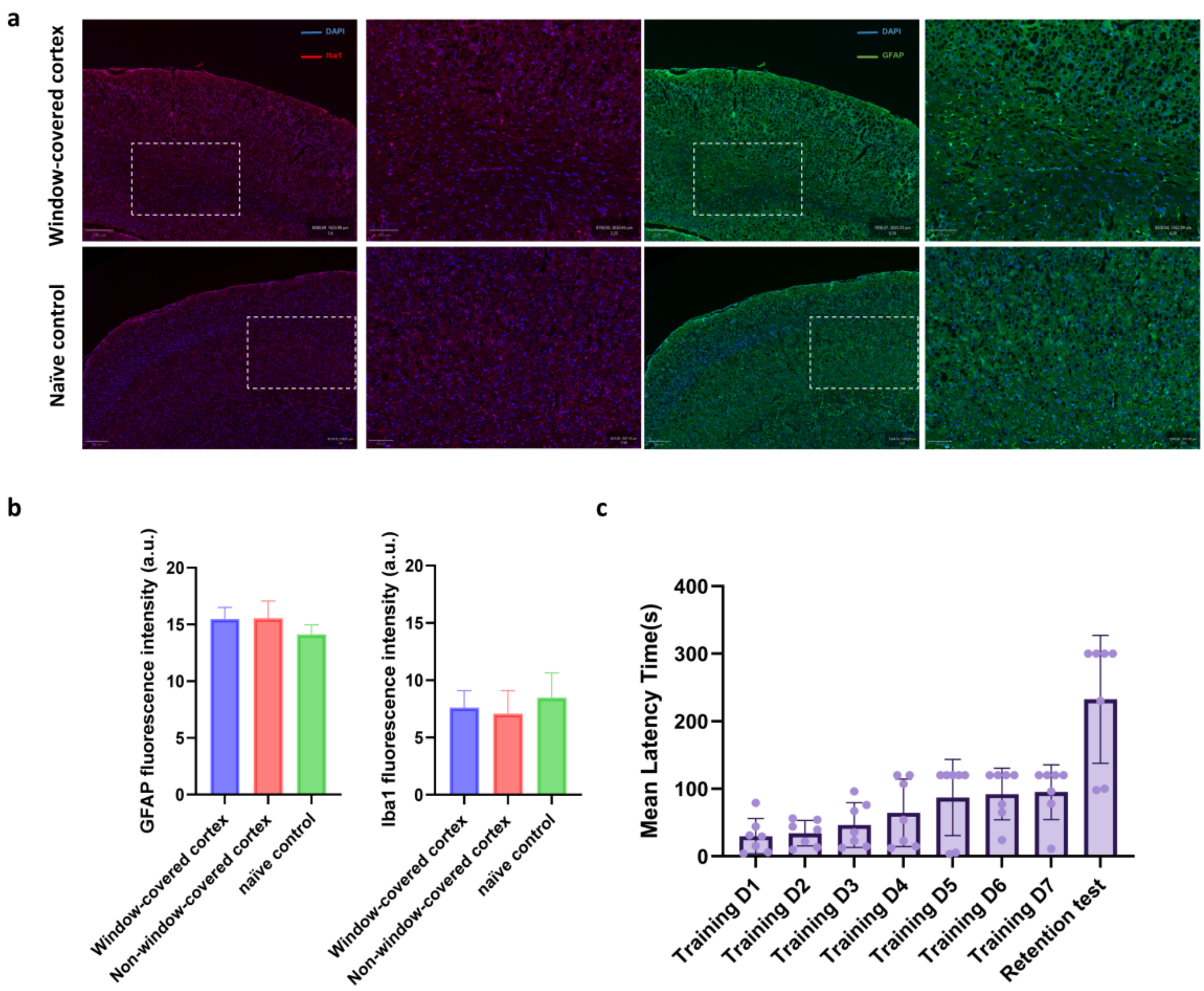

Supplementary Fig. 3. Histological and behavioral assessment of long-term cortical biocompatibility after chronic PMP cranial window implantation

a, Representative confocal images of cortical sections collected after five months of repeated awake ultrasound imaging through the chronic PMP cranial window. Iba1-positive microglia are shown in red, GFAP-positive astrocytes in green, and nuclei are counterstained with DAPI in blue. Images from the window-covered cortex are compared with age-matched naïve control cortex. Dashed boxes indicate the regions shown at higher magnification. No obvious reactive gliosis, microglial accumulation, or focal inflammatory response was observed beneath the chronic window. b, Quantification of background-subtracted GFAP and Iba1 fluorescence intensities in the window-covered cortex, non-window-covered cortex, and naïve control cortex. c, Passive avoidance testing across seven training days and the retention test. Step-through latency increased during training and remained elevated during retention testing, indicating preserved avoidance learning after chronic window implantation and repeated awake imaging.

Data are presented as mean ± SD.

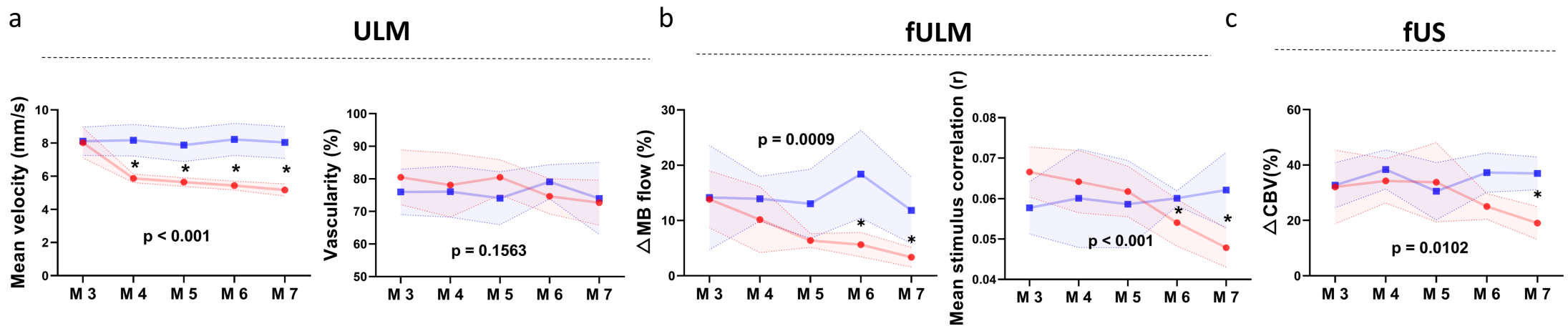

Supplementary Fig. 4. Longitudinal changes in vascular and hemodynamic parameters in 5xFAD mice

a, ULM-derived mean flow velocity and vascularity from M3 to M7. b, fULM-derived ΔMB flow and mean correlation (r) in response to whisker stimulation. c, fUS-derived ΔCBV in response to whisker stimulation. 5xFAD-positive mice are shown as red circles, and negative littermate controls as blue squares. Solid lines represent group means, and shaded regions indicate SD. Asterisks indicate significant differences between genotypes at individual months following Šidák correction for multiple comparisons. n = 7 mice per group.

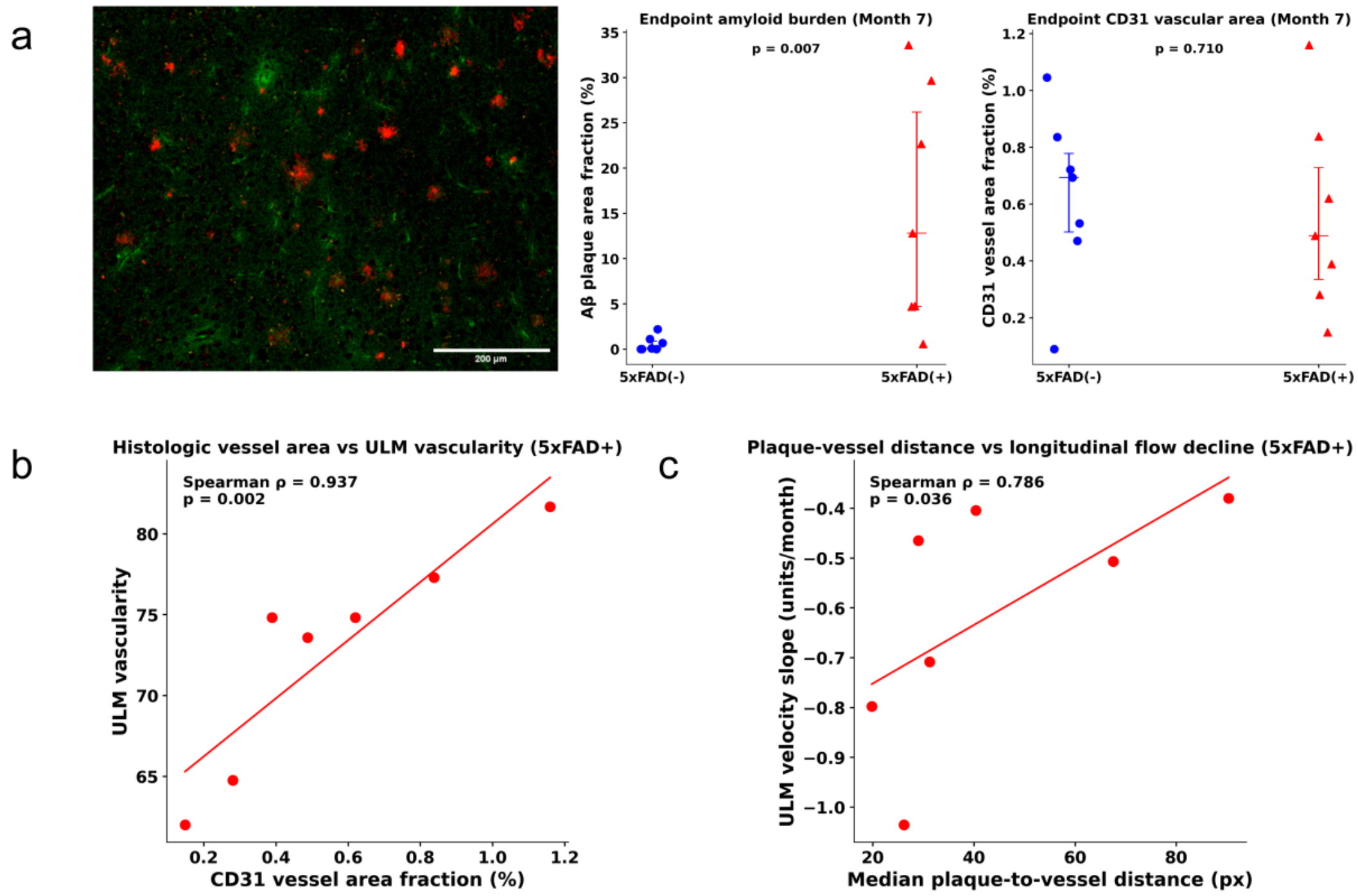

Supplementary Fig. 5. Histological validation of imaging metrics

a, Representative terminal Aβ (red) and CD31 (green) staining; Aβ burden was elevated in 5xFAD(+) mice, while CD31 vessel density did not differ significantly from 5xFAD(−) controls. b, CD31 vessel area fraction correlates with in vivo ULM vascularity in 5xFAD(+) mice. c, Terminal plaque-to-vessel distance correlates with the longitudinal decline in ULM flow velocity.

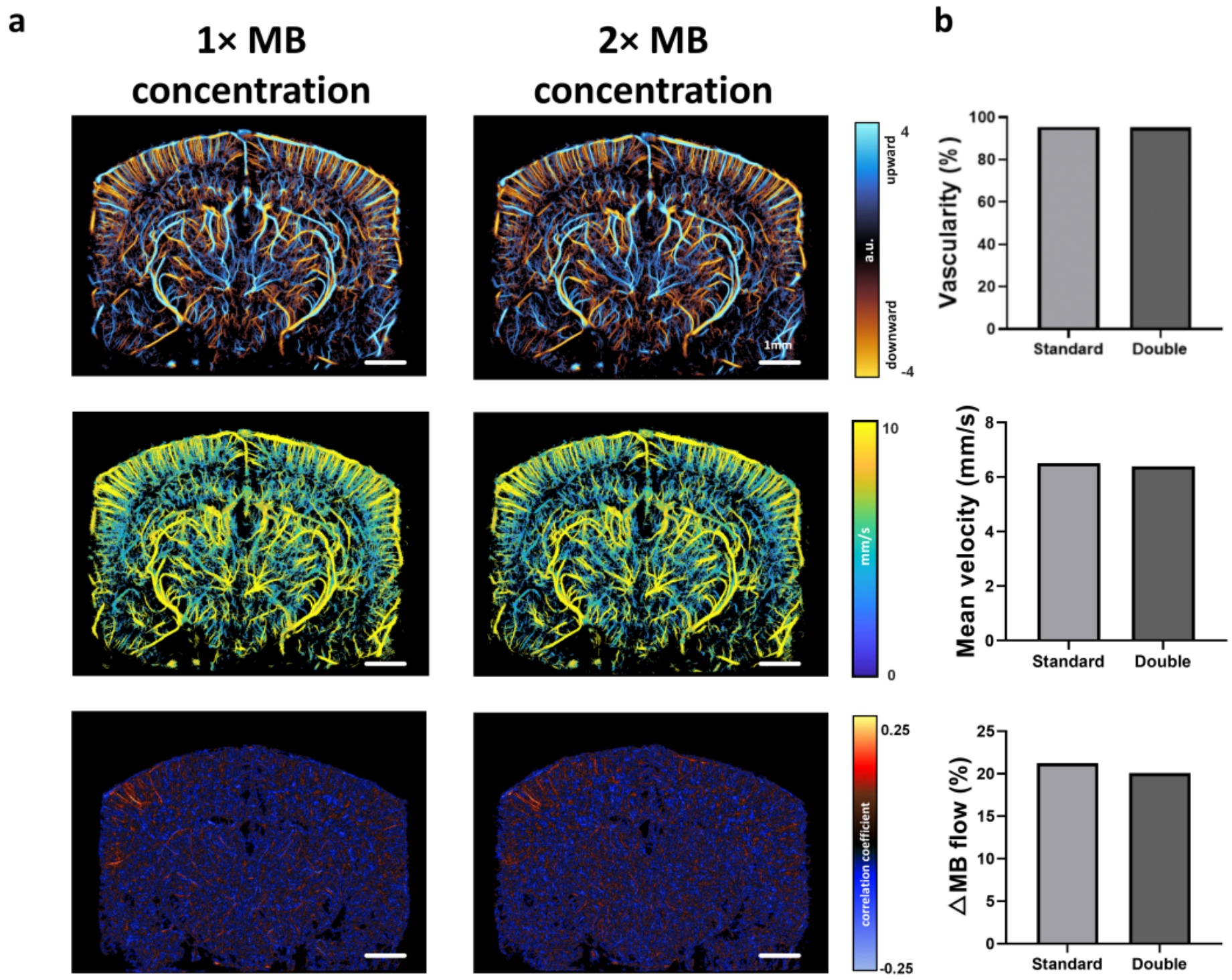

Supplementary Fig. 6. Effect of microbubble concentration on ULM and fULM measurements

a, Representative maps acquired using standard microbubble concentration (1× MB concentration) and double microbubble concentration (2× MB concentration). From top to bottom: ULM vascularity map, ULM velocity map, and fULM activation map.

b, Quantification of vascularity, mean velocity, and fULM ΔMB flow under 1× and 2× MB concentrations, showing minimal differences between the two conditions.

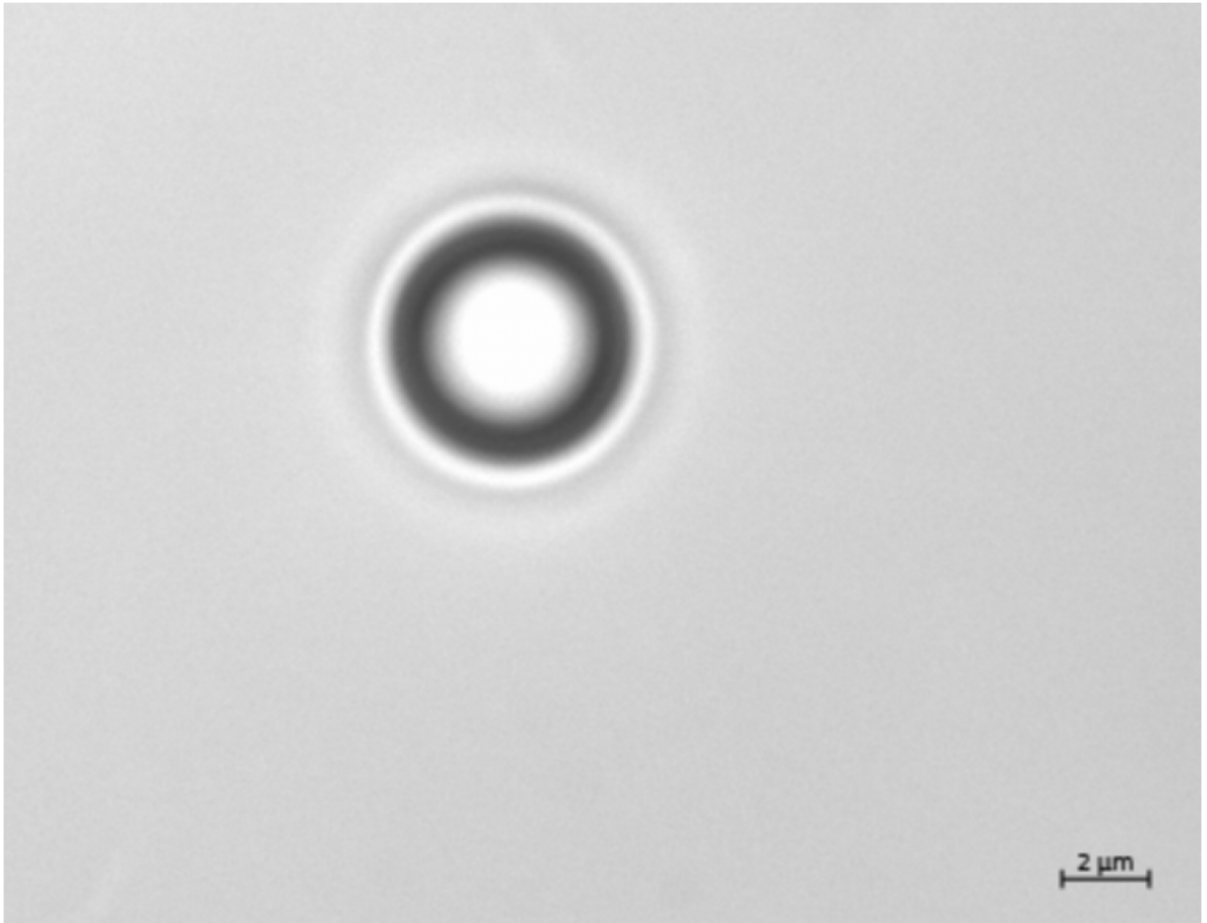

Supplementary Fig. 7. Representative morphology of Lumason® microbubbles

Representative bright-field microscopy image of a freshly reconstituted Lumason® microbubble showing its spherical morphology and well-defined shell structure. Scale bar, 2 µm.

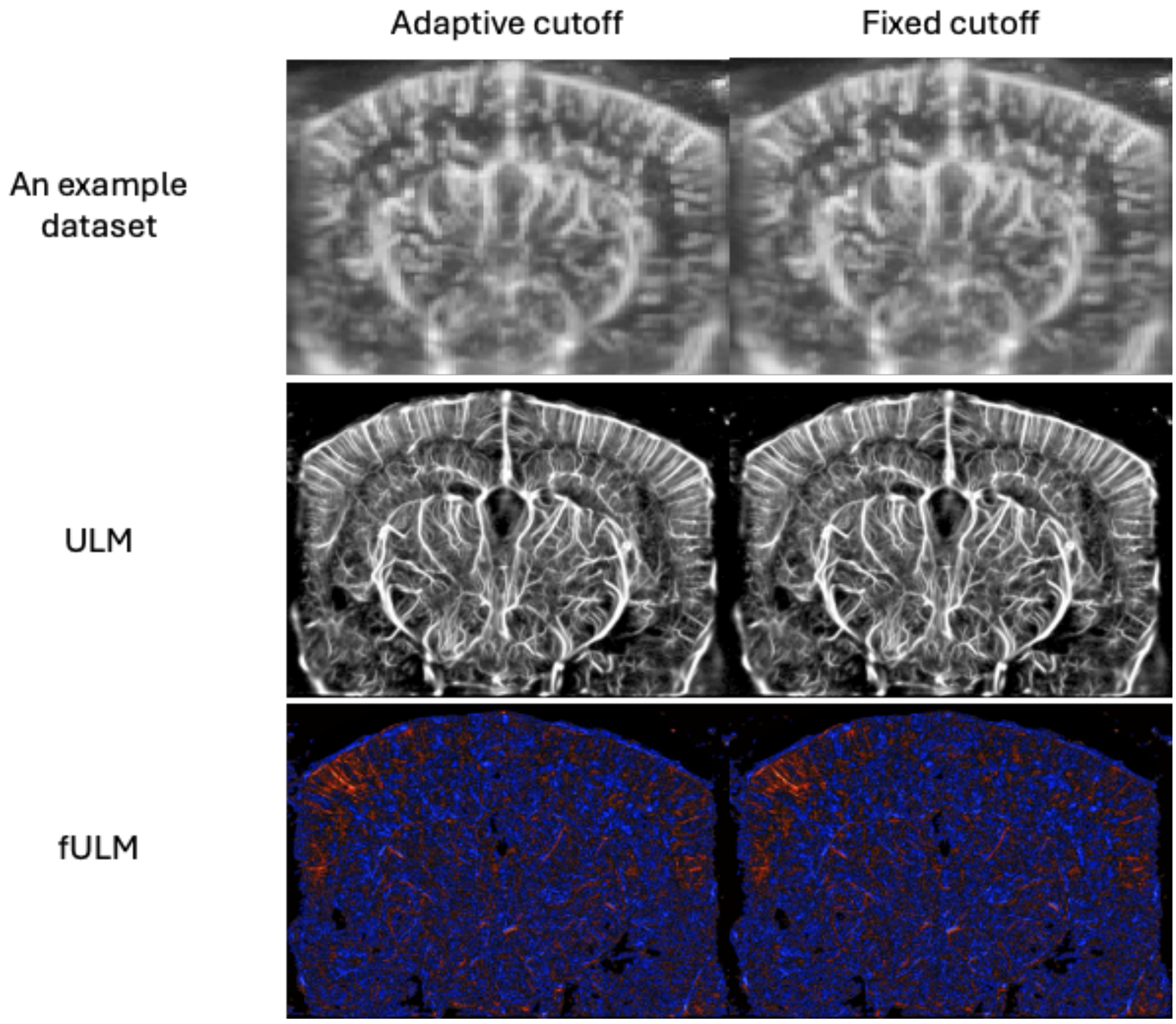

Supplementary Fig. 8. Comparison of adaptive and fixed cutoff settings in a representative dataset

Reconstructions using the adaptive cutoff of 47 (left) and fixed cutoff of 50 (right) produced nearly identical filtered images (top), ULM vascular maps (middle), and fULM maps (bottom), indicating minimal sensitivity to this small cutoff difference after long-duration microbubble accumulation.
